# The role of the opioid system in decision making and cognitive control: A review

**DOI:** 10.3758/s13415-019-00710-6

**Published:** 2019-04-08

**Authors:** Henk van Steenbergen, Marie Eikemo, Siri Leknes

**Affiliations:** 1 Cognitive Psychology Unit, Institute of Psychology, Leiden University, Wassenaarseweg 52, 2333 AK Leiden, The Netherlands; 2 Leiden Institute for Brain and Cognition, Leiden, The Netherlands; 3 Department of Diagnostic Physics, Oslo University Hospital, Oslo, Norway; 4 Department of Psychology, University of Oslo, Oslo, Norway

**Keywords:** Opioid system, Cognitive control, Decision making, Executive function, Value-based choice, Reward, Drugs, Mu-opioid receptors, Affect, Mood, Morphine, Hedonic states

## Abstract

The opioid system regulates affective processing, including pain, pleasure, and reward. Restricting the role of this system to hedonic modulation may be an underestimation, however. Opioid receptors are distributed widely in the human brain, including the more “cognitive” regions in the frontal and parietal lobes. Nonhuman animal research points to opioid modulation of cognitive and decision-making processes. We review emerging evidence on whether acute opioid drug modulation in healthy humans can influence cognitive function, such as how we choose between actions of different values and how we control our behavior in the face of distracting information. Specifically, we review studies employing opioid agonists or antagonists together with experimental paradigms of reward-based decision making, impulsivity, executive functioning, attention, inhibition, and effort. Although this field is still in its infancy, the emerging picture suggests that the mu-opioid system can influence higher-level cognitive function via modulation of valuation, motivation, and control circuits dense in mu-opioid receptors, including orbitofrontal cortex, basal ganglia, amygdalae, anterior cingulate cortex, and prefrontal cortex. The framework that we put forward proposes that opioids influence decision making and cognitive control by increasing the subjective value of reward and reducing aversive arousal. We highlight potential mechanisms that might underlie the effects of mu-opioid signaling on decision making and cognitive control and provide directions for future research.

## Introduction

Pleasure and pain are powerful motivators that determine a great deal of our behavior in daily life. Opioid drugs are known to dampen pain and increase pleasure (Kringelbach & Berridge, 2009; Leknes & Tracey, 2008). The subjective reports of people taking opioids for pain relief or recreation (De Quincey, 2000) have been corroborated by findings that rodents will work to obtain an opioid but also to avoid opioid blockade (Mucha & Iversen, 1984). Accordingly, many influential theories describe the opioid system as the brain’s regulator of affective states (Barbano & Cador, 2007; Berridge & Kringelbach, 2015; Koob & Le Moal, 2001).

Opioid drugs are the “gold standard” treatment for perioperative pain, for example. These drugs also dampen other aversive experiences, such as the sensation of breathlessness (Hayen et al., 2017), psychosocial stress (Bershad, Jaffe, Childs, & de Wit, 2015; Bershad, Miller, Norman, & de Wit, 2018), and depressive symptoms (Peciña et al., 2018). Evidence from nonhuman animal studies highlights the importance of the opioid system in regulating not just aversive experiences but also motivation and “liking” of food (Baldo, 2016; S. Peciña & Smith, 2010), social contact (Loseth, Ellingsen, & Leknes, 2014), and other rewards (Laurent, Morse, & Balleine, 2015). The available human literature is still limited but is suggestive of a similar hedonic regulation by the human opioid system. A recent review of positron emission tomography (PET) studies with opioid receptor-specific tracers posit a central role of the opioid system for positive affective states (Nummenmaa & Tuominen, 2017). Drug studies in both human and nonhuman animals show that blocking opioids reduces food pleasantness and consumption, especially for high-calorie foods (Drewnowski, Krahn, Demitrack, Nairn, & Gosnell, 1992; Eikemo et al., 2016; Price, Christou, Backman, Stone, & Schweinhardt, 2016; Yeomans, 1995; Yeomans & Gray, 2002).

For aversive stimuli, blocking opioid signaling can enhance or maintain responses in aversive learning tasks (Eippert, Bingel, Schoell, Yacubian, & Büchel, 2008; Haaker, Yi, Petrovic, & Olsson, 2017). Opioid blockade can also increase the aversiveness of pain (Anderson, Sheth, Bencherif, Frost, & Campbell, 2002), although this effect is rarely observed with short-lasting experimental pain stimuli (Berna et al., 2018; Eippert et al., 2008; Grevert & Goldstein, 1977). Very recently, studies indicate that social reward processes are similarly modulated by opioids in humans. Indeed, opioid agonist and/or antagonist drugs have been reported to modulate the perceived attractiveness and motivation to view faces of beautiful women (Chelnokova et al., 2014), the relative pleasantness of nude images and frustration at missed opportunity to view these (Buchel, Miedl, & Sprenger, 2018), visual exploration of faces (Chelnokova et al., 2016), and perception of faces with emotional expressions (Bershad, Seiden, & de Wit, 2016; Loseth et al., 2018; Syal et al., 2015; Wardle, Bershad, & de Wit, 2016).

Overall, these findings are in line with the notion that mu-opioid receptor stimulation by endogenous and exogenous opioid peptides causes a shift in valuation along a “hedonic gradient,” ranging from displeasure to pleasure. This shift is not limited to the "liking" of stimuli. Learning and motivation typically increase with increased valuation (Berridge, Robinson, & Aldridge, 2009). Evidence from nonhuman animal studies also shows opioid modulation of learning independently of “liking” responses (Laurent et al., 2015). Moreover, microstimulation with opioid peptides has been shown directly to increase motivation for different reward types in rodents (Mahler & Berridge, 2012) through distinct neural mechanisms (Wassum, Ostlund, Maidment, & Balleine, 2009a).

In rodents, wanting, liking, and reward learning can be modulated by manipulations of opioid receptors in the ventral and dorsal striatum, ventral pallidum, and the central nucleus and basolateral parts of the amygdala (Berridge & Kringelbach, 2015; Wassum, Cely, Balleine, & Maidment, 2011; Wassum, Cely, Maidment, & Balleine, 2009b; Wassum, Ostlund, et al., 2009a). Recently, “hedonic hot- and coldspots” involved in sweet taste “liking” responses also were identified in the rat insula and prefrontal cortices (Castro & Berridge, 2017). Studies using PET imaging and pharmacological MRI in humans suggest that opioids may indeed exert their effects on reward-related behavior through receptors in the orbitofrontal cortex, amygdala, thalamus, insular cortices, ventral and dorsal striatum, and cingulate cortices (Hsu et al., 2013; Love, Stohler, & Zubieta, 2009; Murray et al., 2014; Nummenmaa et al., 2018; Petrovic et al., 2008; Rabiner et al., 2011). A large meta-analysis of fMRI activation associated with subjective value processes showed that the striatum and ventro-medial prefrontal areas (including the orbitofrontal cortex) are key to the valuation process, whereas a different network comprised of the anterior insula, dorsomedial prefrontal cortex, dorsal and posterior striatum, and thalamus may be recruited in response to arousal or salience during valuation (Bartra, McGuire, & Kable, 2013).

**Fig. 1:**
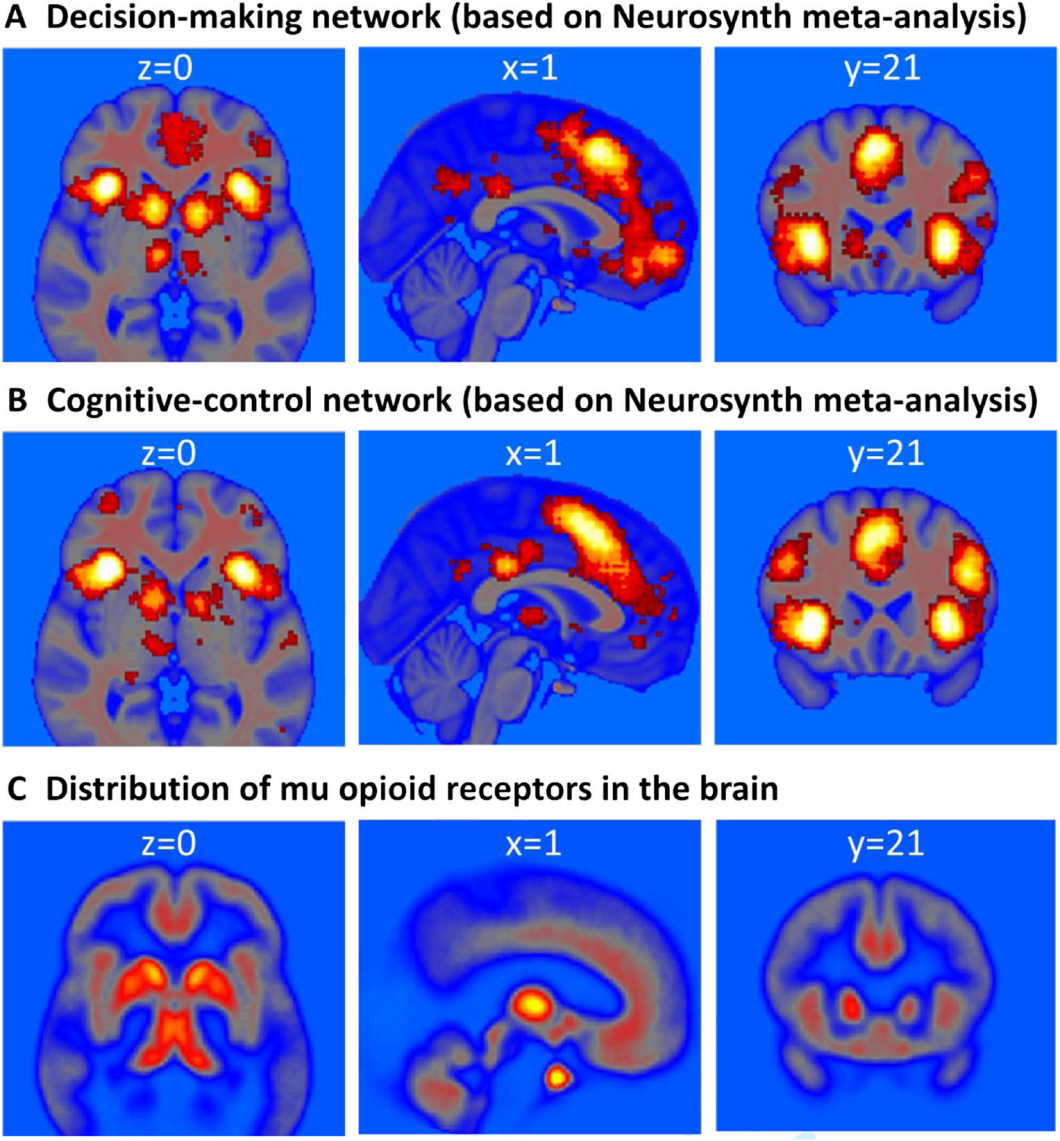
Neural circuits involved in decision making (A) and cognitive control (B) based on meta analyses (analysis date 24 May 2018), showing forward inference maps of statistically significant (false discovery rate, *p* < 0.01) activations in Neurosynth (Yarkoni, Poldrack, Nichols, Van Essen, & Wager, 2011). (C) Density of mu-opioid receptor expression as revealed by [11C]-carfentanil PET (mean nondisplaceable binding potential (BPND) image of 89 PET scans from healthy volunteers; courtesy of Dr. Lauri Nummenmaa). The circuits involved in decision making and cognitive control show particular high density of mu-opioid receptors in the limbic system (including thalamus, basal ganglia, and cingulate cortex) and a moderate density of mu-opioid receptors in cortical regions, such as insular and lateral-prefrontal cortex. Note that mu-opioid receptors regulate many functions. As shown above, receptors are expressed throughout the brain, including in regions less commonly associated with cognition, such as the midbrain (periaqueductal gray), hypothalamus, and cerebellum. These maps and their conjunctions are available at Neurovault (Gorgolewski et al., 2015): https://neurovault.org/collections/4841/

**Fig. 2:**
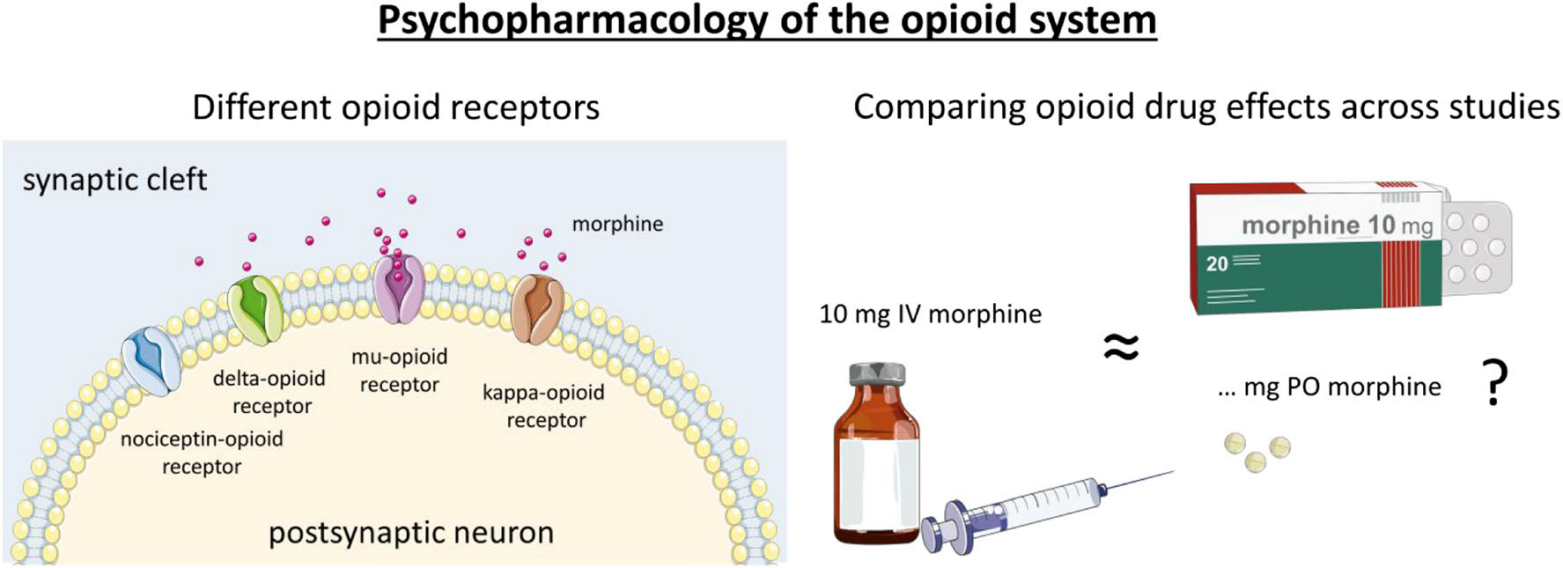
Left panel: Drugs can affect the opioid system via different receptor subtypes. The opioid system is made up of four different opioid receptor types, the mu-, delta, kappa-, and the nociceptin receptors (Corbett, 2009). Several types of endogenous ligands, such as endorphins, enkephalins, dynorphins, endomorphins, and nociceptin activate these (Calo, Guerrini, Rizzi, Salvadori, & Regoli, 2000; Fichna, Janecka, Costentin, & Do Rego, 2007). Drugs, such as morphine and heroin, are considered mu-opioid agonists, i.e., they act primarily on the mu-opioid receptor (Pasternak, 2001). The drugs that block endogenous opioid signaling (antagonists, such as naloxone or naltrexone) in humans typically inhibit activity at both mu- and kappa-receptors. To date, the mechanism of action of the mu-opioid receptor is best understood. Both the analgesic and the euphoric effects of opioid drugs are thought to be mediated by this receptor type (Fields & Margolis, 2015). Although mu-opioid receptors are widely distributed in the brain (and in other parts of the body as well), they are in particular highly expressed in limbic brain areas, such as the basal ganglia, thalamus, and anterior cingulate. They also are expressed to a moderate extent in cortical areas, such as lateral prefrontal and insular regions (Henriksen & Willoch, 2008). Right panel: Mu-opioid receptors are activated by a large number of different drugs, and they are commonly compared in terms of their efficacy to relieve pain at a particular dose and administration method. Opioid drugs are often given as pills (per oral; PO) but also intravenously (IV), transnasally (TN), subcutaneously (SC), and intramuscularly (IM). We have calculated a rough estimate of “morphine equivalence” on the basis of available evidence of analgesic effects. This conversion was primarily based on the values provided in earlier work (Knotkova, Fine, & Portenoy, 2009; Zacny, 1995). It is important to emphasize that dosages with similar analgesic properties can have different effects on cognitive function, and a simple conversion does not capture the different pharmacokinetics of different drugs. To emphasize that the conversion is coarse for the present purposes, we do not report the exact equianalgesic doses of morphine. Instead, we have categorized the dose as a low, medium, or high dose, using 4 mg and 7 mg IV of morphine as the minimal cutoff values for the medium and high dose, respectively. As for the opioid antagonists, these were categorized on the basis of available PET evidence about the proportion of mu-opioid receptors blocked by a given drug dose (Mayberg & Frost, 1990). Doses covering 90-100% of receptors are considered full antagonists (Weerts et al., 2013). Where doses administered were much higher or lower than this range, this is specified in the text. In addition to drugs acting primarily as agonists or antagonist to opioid receptors, the literature also contains several reports from “mixed agonists,” i.e., drugs that act simultaneously as agonists and antagonists at different opioid receptors (Jacob, Michaud, & Tremblay, 1979). This category also covers drugs that act as partial agonists and antagonists, such as buprenorphine, which binds strongly to mu-opioid receptors, but causes less activation of the receptor than the endogenous ligand. Buprenorphine is also a kappa-opioid antagonist. These drugs were also categorized on the basis of their analgesic effects at the doses used in the literature. Figures were produced with graphics from Servier Medical Art (smart.servier.com) under Creative Commons BY 3.0 license.

Recently, rodent researchers have argued convincingly for opioid involvement in choice behaviors beyond the regulation of reinforcement and aversion (Laurent et al., 2015). Indeed, the widespread distribution of opioid receptors throughout the human brain is consistent with a wider role of this neuromodulator for cognition and behavior (Figures 1 and 2). Opioid activation and inhibition also affects other neurotransmitter systems important for cognition, such as dopamine and norepinephrine (Chaijale et al., 2013; Fields & Margolis, 2015; Valentino & Van Bockstaele, 2015)

## Opioid regulation of cognitive control and decision making?

By altering motivational processing and/or learning, opioid drugs could exert profound effects on cognitive control and reward-based decisions even after a single drug administration. Work in rodents indeed shows opioid-induced impairments in some measures of sustained attention and response inhibition (for an up-to-date review see, Jacobson, Wulf, Browne, & Lucki, 2018). Despite decades of nonhuman animal research, much less is known about acute opioid modulation of cognition and decision making in humans. Although a general assumption has been that opioid drugs will impair concentration, human drug studies collecting measures of cognitive control and executive function have typically lacked a strong theoretical motivation for task inclusion.

One recurrent idea in the human literature is that opioids might impair concentration by reducing arousal and discomfort, whereas this same arousal-reducing property might improve cognitive function when participants are tested in stressful situations (Evans & Witt, 1966). Research from the past decade has renewed interest in how arousal can influence cognitive control (Aston-Jones & Cohen, 2005). Some studies have highlighted the specific effects of aversive arousal, i.e., heightened arousal in combination with negative valence (Saunders & Inzlicht, 2015; van Steenbergen, 2015). In models that describe affect as a two-dimensional space with the factors arousal and valence (Russell, 2003), aversive arousal can be conceptualized as a diagonal in the quadrant that combines high levels of arousal with negative valence (Thayer, 1989; Yik, Russell, & Barrett, 1999). Aversive arousal is an integral affective response in many tasks requiring cognitive control (Inzlicht, Bartholow, & Hirsh, 2015). Recent accounts have suggested that aversive arousal tunes goal-directed behavior (Dreisbach & Fischer, 2015; van Steenbergen, Band, & Hommel, 2009) and can be counteracted by the induction of incidental positive affect (van Steenbergen, 2015). Accordingly, it is conceivable that positive affect induced by an opioid drug might downregulate aversive arousal, thereby influencing cognitive control. Such opioid effects would be consistent with emerging work on the stress-relieving properties of opioids (Valentino & Van Bockstaele, 2015).

In the present paper, we present a synthesis of current knowledge of opioid regulation of decision making and the control of goal-directed behavior in the healthy human brain. The studies reviewed have used pharmacological manipulations in healthy humans together with decision-making and cognitive-control tasks. Where possible, we also draw on relevant evidence from PET imaging. Our primary objective is to gain a better understanding of the mechanisms of acute opioid modulation of cognitive processes. We also discuss the possible role of the mu-opioid system in indirect modulation of decision-making and cognitive control via changes in affective states (Braver et al., 2014; Chiew & Braver, 2011; Dreisbach & Goschke, 2004; Isen & Means, 1983; Notebaert & Braem, 2016; van Steenbergen, 2015; Vinckier, Rigoux, Oudiette, & Pessiglione, 2018) and stress (Shields, Sazma, & Yonelinas, 2016). After a description of the methods used for our literature review, we will briefly summarize the main results of the reviewed studies for the domains of decision making and cognitive control. This is followed by an integrative discussion of the reviewed literature in which we put forward a framework that aims to capture the reviewed findings and generate testable hypotheses for future research. Specifically, we propose that opioids influence decision making and cognitive control by increasing the subjective value of reward and reducing aversive arousal. Other avenues for future research are highlighted before we present some general conclusions.

## Literature inclusion

To synthesize the available evidence for acute opioid drug effects on cognition in healthy human volunteers, we searched for studies combining opioid agonist and antagonist drugs with experimental paradigms to investigate decision making, impulsivity, executive functioning, attention, inhibition, and effort. We used Pubmed, Scopus and Google Scholar to search for relevant literature, using a combination of the keyword "opioid" or drug names, such as naltrexone, naloxone, remifentanil, buprenorphine, oxycodone, and morphine and the particular cognitive function (e.g. attention, impulsivity, decision-making). In addition, we included relevant articles cited in recent papers or in earlier reviews by Zacny (1995) and Ersek et al. (2004). Studies were included in this semisystematic review if they were published as an article in a peer-reviewed scientific journal, had tested healthy human volunteers, included a pharmacological manipulation with an opioid agonist and/or antagonist drug, and included one or more paradigms related to the domains of decision making or cognitive control. Both behavioral results and/or neural effects using EEG or fMRI were included in the review. For reasons of clarity and feasibility, papers published before 1957 and studies that measured the relevant drug effects during pain or in combination with another drug were not considered. Beyond these limitations, we strived to include all relevant studies rather than reviewing a subset. Accordingly, we consider our approach a semisystematic review, in the sense that we did not knowingly exclude evidence contrary to (or consistent with) our own opinions about the topic at hand. To compare the different drugs and their doses, we calculated a rough estimate of “morphine equivalence” on the basis of available evidence of analgesic effects (Figure 2).

A quantitative meta-analysis of effects in the reviewed literature was not possible due to 1) variable drug types, doses, and administration methods used across studies, 2) variable tasks and outcome measures reported, and 3) the failure to report means and variance information for relevant outcomes in much of the (earlier) literature. Note that this literature has typically been statistically underpowered, and for topics where both null and positive effects are reported, we give relatively less weight to null findings identified using frequentist statistics only.

## Review of studies on decision-making

### Reward-based decision-making

Only a handful of studies have investigated the behavioral, neural, and psychophysiological responses to reward-based decisions following pharmacological manipulation of the opioid system in healthy humans. In general, these studies are consistent with the available evidence on opioid modulation of rewards presented outside of a decision context. Petrovic and colleagues (Petrovic et al., 2008) used opioid blockade (10 mg IV naloxone, placebo-controlled) to assess the role of endogenous opioids during a gambling task with rewards and losses in 15 healthy men (within-subject). Following naloxone, monetary losses were rated as more aversive and the opioid blockade increased activation in regions such as the ACC and anterior insula. Pleasantness ratings of wins were unaltered by opioid blockade, which nevertheless decreased ACC responses to these rewards. Another study reported no clear effects of opioid blockade (50 mg naltrexone) on BOLD responses to monetary wins and losses (Monetary Incentive Delay task) in 35 healthy participants (Nestor et al., 2017). Very recently, a third fMRI study using naloxone and an incentive delay task with monetary gain and erotic images in 21 healthy men (within-subject) found that compared with placebo, opioid blockade decreased the relative pleasantness of both types of high-value rewards but with a significantly stronger effect on erotic images (Buchel et al., 2018). The authors observed reduced BOLD response to erotic images in bilateral striatum, orbitofrontal cortex, the amygdalae, prefrontal cortex, and hypothalamus but no reduced response to (symbolic) receipt of monetary gains. During the anticipation phase, a small reduction in medial prefrontal cortex and right lateral orbitofrontal cortex activity was observed during cues signaling potential monetary gains (but not erotic images).

Thus, initial evidence of opioid modulation of pleasantness and fMRI responses to rewarded decisions in humans is modest but consistent with the evidence that opioids promote the pleasantness of rewards received outside of a decision context (Chelnokova et al., 2014, 2016; Drewnowski et al., 1992; Eikemo et al., 2016; Murray et al., 2014; Price et al., 2016; Yeomans, 1995; Yeomans & Gray, 2002). Whether opioid agonist drugs increase the liking of choice outcomes in humans remains to be seen.

### Reward learning and motivation in decision making

What about opioid modulation of reward learning and motivation in humans? Studies using measures of motivation and learning have shown more consistent opioid modulations than those on reward responses. Using a Pavlovian instrumental transfer (PIT) task with chocolate reinforcement, Weber et al. (2016) found a significant reduction in the difference between button presses for rewarded and unrewarded stimuli following 50 mg naltrexone (n = 40) compared with placebo (n = 40), indicating reduced motivation to exert effort for rewards after opioid blockade. Eikemo et al. (2017) tested the effects of 50 mg of naltrexone, 10 mg of morphine, and placebo on rewarded choice in healthy men (n = 30, within-subjects). Using a drift diffusion model, they found that mu-opioid agonism enhanced and blockade decreased, processing efficiency in the reward task, which they interpreted as a measure of motivation to obtain reward. Morphine also increased the bias towards the high-value response option (associated with high reward probability). Using opioid blockade (50 mg of naltrexone, n = 21) and placebo (n = 20) before a slot-machine task and roulette task, Porchet et al. (2013) found that naltrexone moderately attenuated the motivational ratings after near-misses compared with placebo. However, contrary to the above reviewed findings, participants under opioid blockade also exhibited signs of increased reward sensitivity (a larger change in the skin conductance response to wins and higher confidence following winning-streaks). Finally, a study of opioid effects on financial decisions (trading game) suggested that opioid blockade (naltrexone 50 mg, n = 62) decreases reinforcement learning compared with placebo (n = 116) in healthy participants (Efremidze, Sarraf, Miotto, & Zak, 2017).

In summary, the available evidence provides some support for involvement of the endogenous opioid system in value-based decision making, suggesting that blocking opioid receptors may reduce, and stimulating receptors may enhance, the motivational value of and learning about high-value stimuli and choices for these options. However, pharmacological studies in healthy humans are still scarce, and results are not homogenous. So far, the opioid effects on value-based decision making appear broadly consistent with the extensive evidence from rodent research (Berridge & Kringelbach, 2015; Laurent et al., 2015; Lutz & Kieffer, 2013), but more research into this area in humans is needed.

### Impulsive choices

Impulsivity is a broad construct related to impulsive choice (as measured by probability discounting, gambling tasks, and delay discounting) and impulsive action (e.g., failure to inhibit prepotent responses, see *Inhibition and Effort*). Correlations between trait impulsivity and performance on tasks measuring impulsive choice or action are low, as are correlations of performance between these tasks. Nevertheless, trait impulsivity has been related to opioid receptor binding in a PET study. Specifically, NEO Personality Inventory (Costa & McCrae, 1992) measures related to lack of control over cravings or desires, correlated with receptor-binding potential in medial frontal cortex, nucleus accumbens/ventral pallidum, and the right amygdala in 19 young males (Love et al., 2009).

A handful of opioid drug studies have investigated impulsive choices using delay discounting tasks where subjects choose between smaller immediate rewards or larger delayed rewards. Two initial studies reported no significant effect of naltrexone (50 mg PO) on impulsive choice ratio in nine (Mitchell, Tavares, Fields, D’Esposito, & Boettiger, 2007) and ten healthy controls (Boettiger, Kelley, Mitchell, D’Esposito, & Fields, 2009). In a larger study by Weber et al. (2016), a trend towards reduced impulsive choice was reported in 40 healthy people receiving naltrexone (50 mg) compared with a placebo group of the same size. Few studies report effects of opioid *agonism* on impulsivity measures in healthy humans. In Zacny and de Wit (2009), a battery of five tasks measuring aspects of choice and motor impulsivity was administered following three separate doses of oxycodone (5, 10, or 20 mg PO) compared with placebo (within-subject; n = 12). They did not find any significant effect on any of the tasks, including delay discounting, even at the higher oxycodone doses where participants reported feeling the drug effects. Furthermore, Eikemo et al. (2017) found no credible effects of either 50 mg of naltrexone or 10 mg of morphine on reward-related impulsivity as measured by speed-accuracy trade-off in a probabilistic reward task.

Together, these results indicate that blocking the majority (>90%) of the μ-opioid receptors in the brain using 50 mg of naltrexone (Weerts et al., 2013) does not cause a large reduction in measures of impulsive reward choices in healthy humans. Rodent work similarly indicates limited or no effects of opioid blockade on tests of impulsive behavior (Kieres et al., 2004; Pattij, Schetters, Janssen, Wiskerke, & Schoffelmeer, 2009). For opioid agonism, the preliminary evidence in humans is at odds with rodent findings that acute opioid administration increases impulsivity.

## Review of studies on cognitive control

### Neuropsychological tests of executive functions

The nonhuman animal literature yields minimal information about opioid modulation of executive function. These functions are typically impaired in opioid dependence, but this impairment could be related to other factors and mechanisms than opioid receptor functioning. Indeed, working memory training was shown to increase future orientation and decrease delay discounting in opioid-dependent individuals (Bickel, Yi, Landes, Hill, & Baxter, 2011). While the little evidence available from opioid antagonist studies do not suggest a central role of the endogenous opioid system in executive function, studies employing acute doses of opioid agonists do report modulation of executive functions.

Most consistent evidence for an impact of opioid drugs on executive function comes from studies implementing the digit symbol substitution task (DSST, also known as the coding task), which requires participants to substitute symbols and digits using a particular key. The DSST is the most commonly included task in opioid administration studies. A speeded subtest of the Wechsler Adult Intelligence Scale, it is designed to measure functions related to “processing speed” (Wechsler, 2014). However, recent work has shown that the DSST task does not asses basic psychomotor speed, but instead reflects a mixture of executive functioning processes including inhibition, shifting, and updating (Knowles et al., 2015).

**Fig. 3:**
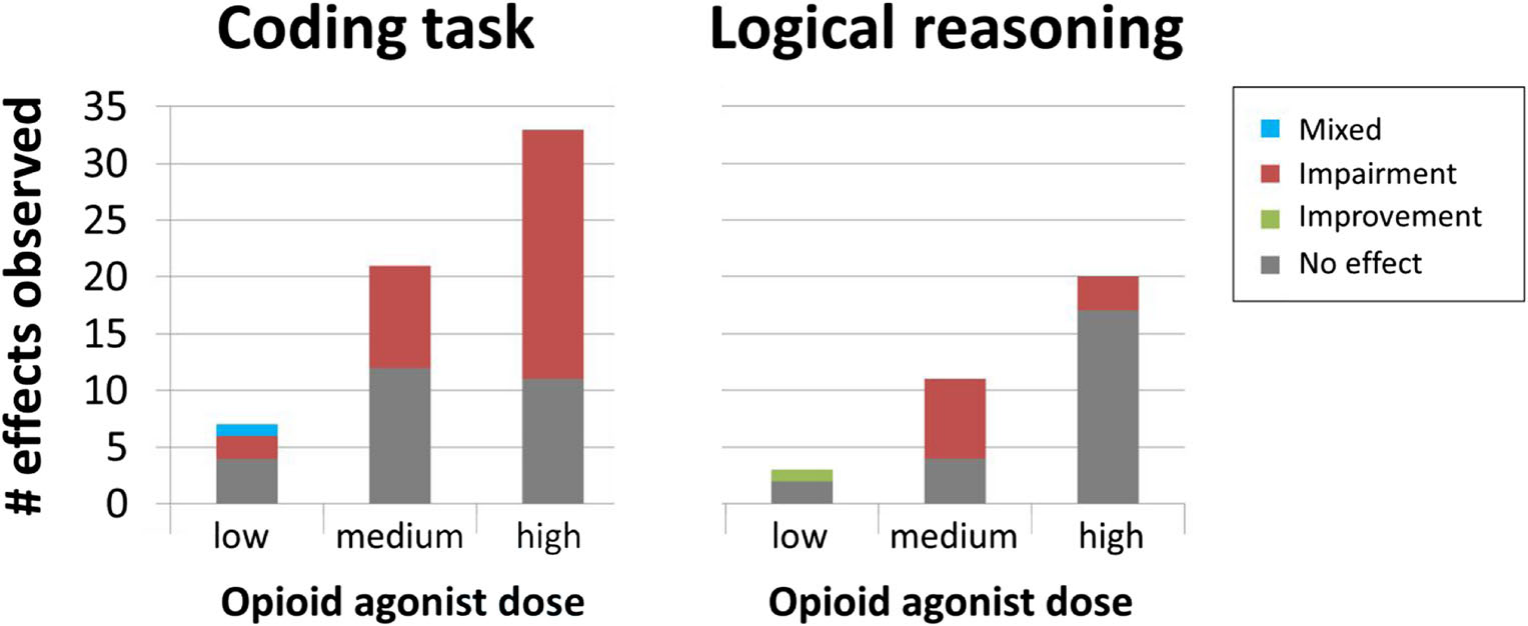
The number of times opioid agonist drug has shown a significant impairment on coding task (DSST) performance and logical reasoning for all type of drugs used in the studies (antagonist drug effects not included, see details in Tables 1 and 2). Dose refers to the minimal dose needed to produce a significant effect; if no effect was observed, we used the maximum dose used in the study.

An early study reported that a low dose of the opioid agonist codeine impairs coding performance in medical troops tested at 2,000 feet (610 meters) altitude but improved performance when they performed this test at 15,000 feet (4,572 meters) elevation (Evans & Witt, 1966). Few subsequent studies have assessed context-sensitive effects of opioids on cognition. Instead most studies have simply included DSST performance as part of a cognitive test battery in a standard psychopharmacological protocol with no systematic manipulation of context. More than 30 such studies have investigated the effects of various types of opioid agonists. The majority of these studies have observed coding impairments (Table 1). Two studies using opioid antagonists reported no effect on coding performance (File & Silverstone, 1981; Zacny, Coalson, Lichtor, Yajnik, & Thapar, 1994a). As Figure 3 (left panel) shows, most performance impairments have been reported in studies using high doses of opioid agonists only, although some studies have observed performance decrements at medium doses (e.g., hydrocodone, oxycodone, and partial agonists). This suggests that the effect of opioid agonists on coding performance might be dose-related, even though a clear link between plasma concentrations and DSST impairments is currently lacking (Strand, Arnestad, Fjeld, & Mørland, 2017).

**Table 1:** Pharmacological studies reporting effect on coding test

Reference	Sample	Drug type	Drug	Route	Dose	Max dose1	Effect2	Effect dose3
Kornetsky, Humphries, & Evarts, 1957	4F/6M	Agonist	Meperidine	PO	50, 100 mg	low	...	_
Smith, Semke, & Beecher, 1962 (Study 1)	24M	Agonist	Morphine	SC	10 mg	high	↓	high
Smith, Semke, & Beecher, 1962 (Study 2)	24M	Agonist	Heroin	SC	4 mg	high	↓	high
		Agonist	Morphine	SC	10 mg	high	↓	high
Evans & Witt, 1966	16M	Agonist	Codeine	PO	32 mg	low	↕	_
File & Silverstone, 1981	6F/6M	Antagonist	Naloxone	IV	0.8, 1.6 mg	-low	...	_
Jarvik, Simpson, Guthrie, & Liston, 1981	20M	Agonist	Morphine	IM	10 mg	high	...	_
Redpath & Pleuvry, 1982	4F/6M	Agonist	Codeine	PO	30, 60 mg	low	...	_
Bradley & Nicholson, 1986	6F	Agonist	Codeine	PO	30, 90 mg	med	...	_
Saarialho-Kere, Mattila, & Seppälä, 1986	10F/M	Agonist	Codeine	PO	100 mg	med	...	_
		Mixed agonist	Pentazocine	PO	75 mg	med	...	_
Bradley & Nicholson, 1987	7F	Mixed agonist	Meptazinol	PO	100, 200 mg	high	...	_
		Mixed agonist	Pentazocine	PO	0, 25, 50 mg	low	↓	low
Saarialho-Kere, Mattila, Paloheimo, & Seppälä, 1987	12F/M	Mixed agonist	Buprenorphine	SC	0.4 mg	high	↓	high
Saarialho-Kere, 1988	5F/7M	Mixed agonist	Nalbuphine	IM	0-10.5 mg	high	↓	high
Saarialho-Kere, Mattila, & Seppälä, 1988	11F/M	Mixed agonist	Pentazocine	IM	30 mg	med	↓	med
MacDonald, Gough, Nicoll, & Dow, 1989	12M	Mixed agonist	Buprenorphine	IM	0.3 mg	high	↓	high
Saarialho-Kere, Mattila, & Seppälä, 1989	9F/M	Agonist	Oxycodone	IM	9.1 mg	high	...	_
Zacny, Lichtor, & de Wit, 1992	6M	Agonist	Fentanyl	IV	0.050 mg	med	...	_
Zacny et al., 1993	1F/9M	Agonist	Meperidine	IV	17.5-70 mg	high	...	_
Oliveto, Bickel, Kamien, Hughes, & Higgins, 1994	2F/7M	Agonist	Hydromorphone	PO	1-6 mg	high	...	_
Veselis et al. 1994	1F/2M versus 3F/3M	Agonist	Fentanyl	IV	Continuous infusion: 1, 1.5, 2.5 ng/ml	high	↓	med
Zacny, Coalson, Lichtor, Yajnik, & Thapar, 1994c (Study 1)	3F/6M	Antagonist	Naloxone	IV	0, 0.01, 0.1, 1.0 mg	-low	...	_
Zacny, Coalson, Lichtor, Yajnik, & Thapar, 1994a (Study 2)	3F/5M	Antagonist	Naloxone	IV	0, 1.0, 3.0, 10 mg	-med	...	_
Zacny, Lichtor, Flemming, Coalson, & Thompson, 1994b	2F/10M	Agonist	Morphine	IV	2.5-10 mg	high	↓	high
Zacny, Lichtor, Thapar, et al., 1994c	5F/7M	Agonist	Morphine	IV	10 mg	high	...	_
		Partial Agonist	Butorphanol	IV	0.5, 1, 2 mg	high	↓	high
Zacny, Lichtor, Klafta, Alessi, & Apfelbaum, 1996a	3F/7M	Agonist	Codeine	PO	60 mg Codeine/600 mg Acetaminophen	low	...	_
		Mixed agonist	Butorphanol	TN	1, 2 mg	high	↓	high
Zacny, Conley, & Galinkin, 1997a	5F/11M	Agonist	Morphine	IV	10 mg	high	...	_
		Mixed agonist	Buprenorphine	IV	0.075, 0.15, 0.3	high	↓	low
Zacny, Conley, & Marks, 1997b	4F/12M	Agonist	Morphine	IV	10 mg	high	...	_
		Mixed agonist	Nalbuphine	IV	2.5, 5, 10 mg	high	↓	high
Walker & Zacny, 1998	3F/9M	Agonist	Codeine	PO	60, 120 mg	med	...	_
		Agonist	Morphine	PO	20, 40 mg	med	...	_
Zacny, Hill, Black, & Sadeghi, 1998	15F/2M	Agonist	Hydromorphone	IV	0.33, 0.65, 1.3 mg	high	↓	high
		Agonist	Morphine	IV	5, 10 mg	high	...	_
Black, Hill, & Zacny, 1999	2F/8M	Agonist	Alfentanil	IV	16, 32, 64 ng/ml	high	↓	high
		Agonist	Remifentanil	IV	Continuous infusion: 0.75, 1.5, 3 ng/ml	high	↓	med
Walker & Zacny, 1999	6F/10M	Agonist	Hydromorphone	IV	Cumulative injections of: .33, .65, 1.3 mg	high	↓	high
		Agonist	Meperidine	IV	Cumulative injections of: 17.5, 35, 70 mg	high	↓	high
		Agonist	Morphine	IV	Cumulative injections of: 2.5, 5, 10 mg	high	...	_
Hill & Zacny, 2000	5F/12M	Agonist	Hydromorphine	IV	Cumulative injections of .33, .65, 1.3 mg	high	↓	high
		Agonist	Morphine	IV	Cumulative injections of 5, 10 mg	high	...	_
Marsch et al., 2001	18M	Agonist	Morphine	IV	Three infusion rates (2 min bolus, 15 min, and 60 min) of 5, 10 mg	high	↓	high
Walker, Zacny, Galva, & Lichtor, 2001	5F/10M	Agonist	Morphine	IV	2.5, 5, 10 mg	high	↓	high
		Mixed agonist	Butorphanol	IV	0.5, 1, 2 mg	med	↓	med
		Mixed agonist	Nalbuphine	IV	Cumulative injections of 2.5, 5, 10 mg	high	↓	high
		Mixed agonist	Pentazocine	IV	7.5, 15 mg	low	...	_
Zacny & Gutierrez, 2003	9F/9M	Agonist	Morphine	PO	40 mg	med	...	_
		Agonist	Oxycodone	PO	10 mg, 20 mg, 30 mg	high	↓	high
Zacny, 2003	9F/9M	Agonist	Hydrocodone	PO	5 mg Hydrocodone/1.5 mg Homatropine, 10 mg Hydrocodone/3 mg Homatropine, 20 mg Hydrocodone/6 mg Homatropine	med	↓	med
		Agonist	Morphine	PO	40 mg	med	...	_
Zacny & Goldman, 2004	9F/9M	Agonist	Morphine	PO	40 mg	med	...	_
		Agonist	Propoxyphene napsylate	PO	50, 100, 200 mg	med	...	_
Escher et al., 2007	12M	Mixed agonist	Buprenorphine	IV	0.15 mg	med	↓	med
Zacny & Gutierrez, 2008	8F/8M	Agonist	Hydrocodone	PO	5 mg Hydrocodone/325 mg Acetaminophen, 10 mg Hydrocodone/650 mg Acetaminophen	med	...	_
		Agonist	Oxycodone	PO	5 mg Oxycodone/325 mg Acetaminophen, 10 mg Oxycodone/650 mg Acetaminophen	med	↓	med
Zacny & Lichtor, 2008	10F/10M	Agonist	Morphine	PO	30, 60 mg	high	↓	high
		Agonist	Oxycodone	PO	10, 20 mg	high	↓	high
Cherrier, Amory, Ersek, Risler, & Shen, 2009	40F/31M	Agonist	Oxycodone	PO	10 mg	med	↓	med
Zacny & Gutierrez, 2009	10F/10M	Agonist	Hydrocodone	PO	15 mg Hydrocodone/487 mg Acetaminophen, 30 mg Hydrocodone/975 mg Acetaminophen	high	↓	high
		Agonist	Oxycodone	PO	10 mg Oxycodone/487 mg Acetaminophen, 20 mg Oxycodone/975 mg Acetaminphen	high	↓	med
Zacny & Gutierrez, 2011	6F/8M	Agonist	Oxycodone	PO	10 mg	med	...	_

F = female; M = male participants. Studies used within-subject designs unless otherwise noted.

^1^Estimated dose for agonists based on morphine equivalent of the maximum dose of in the respective study. For antagonist drugs, the estimated dose is preceded by a minus. See caption of Figure 2 for more details.

^2^Arrow indicates significantly improved (↑) , impaired (↓), mixed effects (↕) or null-effects (…) on performance relative to a placebo control condition.

^3^Minimum estimated dose at which the indicated effect is significantly different from placebo.

Another commonly investigated function in the context of opioid drugs is logical reasoning. Effects of opioid agonists on a logical reasoning task were first reported by Evans and Smith (1964) who observed that a low dose of morphine in four participants improved performance on a test assessing logical judgements compared with four placebo-treated people. However, the majority of subsequent studies in larger samples have failed to replicate this effect with low opioid drug doses (Table 2), although some impairments in logical reasoning have been observed with medium and high doses of full and mixed agonists (Figure 3, right panel). We are not aware of studies that have reported the effects of opioid antagonists on logical reasoning.

**Table 2:** Pharmacological studies reporting effect on logical reasoning

Reference	Sample	Drug type	Drug	Route	Dose	Max dose1	Effect2	Effect dose3
Evans & Smith, 1964	4F/M versus 4F/M	Agonist	Morphine	PO	16 mg	low	↑	low
Zacny, Lichtor, Thapar, et al., 1994a	5F/7M	Agonist	Morphine	IV	10 mg	high	...	_
		Partial Agonist	Butorphanol	IV	0.5, 1, 2 mg	high	...	_
Zacny, Lichtor, Klafta, Alessi, & Apfelbaum, 1996a	3F/7M	Agonist	Codeine	PO	60 mg Codeine/600 mg Acetaminophen	low	...	_
		Mixed agonist	Butorphanol	TN	1, 2 mg	high	...	_
Zacny, Conley, & Galinkin, 1997a	5F/11M	Agonist	Morphine	IV	10 mg	high	...	_
		Mixed agonist	Buprenorphine	IV	0, 0.075, 0.15, 0.3	high	↓	high
Zacny, Conley, & Marks, 1997b	4F/12M	Agonist	Morphine	IV	10 mg	high	...	_
		Mixed agonist	Nalbuphine	IV	2.5, 5, 10 mg	high	...	_
Walker & Zacny, 1998	3F/9M	Agonist	Codeine	PO	60, 120 mg	med	...	_
		Agonist	Morphine	PO	20, 40 mg	med	...	_
Zacny, Hill, Black, & Sadeghi, 1998	15F/2M	Agonist	Hydromorphone	IV	0.33, 0.65, 1.3 mg	high	...	_
		Agonist	Morphine	IV	5, 10 mg	high	...	_
Walker & Zacny, 1999	6F/10M	Agonist	Hydromorphone	IV	Cumulative injections of: .33, .65, 1.3 mg	high	...	_
		Agonist	Meperidine	IV	Cumulative injections of: 17.5, 35, 70 mg	high	...	_
		Agonist	Morphine	IV	Cumulative injections of: 2.5, 5, 10 mg	high	...	_
Hill & Zacny, 2000	5F/12M	Agonist	Hydromorphine	IV	Cumulative injections of .33, .65, 1.3 mg	high	...	_
		Agonist	Morphine	IV	Cumulative injections of 5, 10 mg	high	...	_
Marsch et al., 2001	18M	Agonist	Morphine	IV	Three infusion rates (2 min bolus, 15 min, and 60 min) of 5, 10 mg	high	...	_
Walker, Zacny, Galva, & Lichtor, 2001	5F/10M	Agonist	Morphine	IV	2.5, 5, 10 mg	high	...	_
		Mixed agonist	Butorphanol	IV	0.5, 1, 2 mg	med	↓	med
		Mixed agonist	Nalbuphine	IV	Cumulative injections of 2.5, 5, 10 mg	high	...	_
		Mixed agonist	Pentazocine	IV	7.5, 15 mg	low	...	_
Zacny & Gutierrez, 2003	9F/9M	Agonist	Morphine	PO	40 mg	med	...	_
		Agonist	Oxycodone	PO	10, 20, 30 mg Oxycodone	high	...	_
Zacny, 2003	9F/9M	Agonist	Hydrocodone	PO	5 mg Hydrocodone/1.5 mg Homatropine, 10 mg Hydrocodone/3 mg Homatropine, 20 mg Hydrocodone/6 mg Homatropine	med	↓	med
		Agonist	Morphine	PO	40 mg	med	↓	med
Zacny & Gutierrez, 2008	8F/8M	Agonist	Hydrocodone	PO	5 mg Hydrocodone/325 mg Acetaminophen, 10 mg Hydrocodone/650 mg Acetaminophen	med	↓	med
		Agonist	Oxycodone	PO	5 mg Oxycodone/325 mg Acetaminophen, 10 mg Oxycodone/650 mg Acetaminophen	med	↓	med
Zacny & Lichtor, 2008	10F/10M	Agonist	Morphine	PO	30, 60 mg	high	↓	high
		Agonist	Oxycodone	PO	10, 20 mg	high	↓	high
Zacny & Gutierrez, 2009	10F/10M	Agonist	Hydrocodone	PO	15 mg Hydrocodone/487 mg Acetaminophen, 30mg Hydrocodone/975mg Acetaminophen	high	↓	med
		Agonist	Oxycodone	PO	10 mg Oxycodone/487 mg Acetaminophen, 20 mg Oxycodone/975 mg Acetaminophen	high	↓	med
Zacny & Gutierrez, 2011	6F/8M	Agonist	Oxycodone	PO	10 mg	med	...	_

F = female; M = male participants. Studies used within-subject designs unless otherwise noted.

^1^Estimated dose for agonists based on morphine equivalent of the maximum dose of in the respective study. For antagonist drugs, the estimated dose is preceded by a minus. See caption of Figure 2 for more details.

^2^Arrow indicates significantly improved (↑) , impaired (↓), mixed effects (↕) or null-effects (…) on performance relative to a placebo control condition.

^3^Minimum estimated dose at which the indicated effect is significantly different from placebo.

Working memory is a central aspect of executive functioning that is well known to be modulated by catecholamine systems that directly modulate prefrontal brain activity (Robbins & Arnsten, 2009). Interestingly, across a wide variety of doses and drugs in 16 studies, opioid agonists and antagonists typically do not affect working memory performance (Table 3). Three studies did observe effects on working memory (Ghoneim, Mewaldt, & Thatcher, 1975; Martín del Campo, McMurray, Besser, & Grossman, 1992; Székely, Török, Karczag, Tolna, & Till, 1986) but showed findings in opposite directions and had small study samples (8 or 10 males per study).

**Table 3:** Pharmacological studies reporting effect on working memory

Reference	Sample	Drug type	Drug	Route	Dose	Max dose1	Effect2	Effect dose3
Evans & Smith, 1964	4F/M versus 4F/M	Agonist	Morphine	PO	16 mg	low	...	_
Ghoneim, Mewaldt, & Thatcher, 1975	10M	Agonist	Fentanyl	IV	0.1, 0.2 mg	high	↓	high
		Agonist	Fentanyl	IV	0.1, 0.2 mg	high	...	_
Volavka, Dornbush, Mallya, & Cho, 1979	26M	Antagonist	Naloxone	IV	10, 20 mg	-med	...	_
File & Silverstone, 1981	6F/6M	Antagonist	Naloxone	IV	0.8, 1.6 mg	-low	...	_
Cohen, Murphy, Cohen, Weingartner, & Pickar, 1983	3F/4M	Antagonist	Naloxone	IV	21, 70, 140 mg	-high	...	_
Székely, Török, Karczag, Tolna, & Till, 1986	8M	Agonist	Dihydrocodeine	SC	20 mg	low	↑	low
Saarialho-Kere, Mattila, & Seppälä, 1988	11F/M	Mixed agonist	Pentazocine	IM	30 mg	med	...	_
MacDonald, Gough, Nicoll, & Dow, 1989	12M	Mixed agonist	Buprenorphine	IM	0.3 mg	high	...	_
Martín del Campo, McMurray, Besser, & Grossman, 1992	8M	Antagonist	Naloxone	IV	Cumulative infusion: 10-mg bolus of Naloxone was given as a rapid bolus, followed by an infusion of 7 mg/hr for 12 hr	-med	↓	-med
Hanks, O’Neill, Simpson, Wesnes, & A, 1995	4F/8M	Agonist	Morphine	PO	10, 15 mg	low	...	_
Zacny, Lichtor, Klafta, Alessi, & Apfelbaum, 1996a	3F/7M	Agonist	Codeine	PO	60 mg Codeine/600 mg Acetaminophen	low	...	_
		Mixed agonist	Butorphanol	TN	1, 2 mg	high	...	_
Black, Hill, & Zacny, 1999	2F/8M	Agonist	Alfentanil	IV	16, 32, 64 ng/ml	high	...	_
		Agonist	Remifentanil	IV	Continuous infusion: 0.75, 1.5, 3 ng/ml	high	...	_
Zacny & Goldman, 2004	9F/9M	Agonist	Morphine	PO	40 mg	med	...	_
		Agonist	Propoxyphene napsylate	PO	50, 100, 200 mg	med	...	_
Verster, Veldhuijzen, & Volkerts, 2006	12F/6M	Agonist	Oxycodone	PO	5 mg Oxycodone/325 mg Acetaminophen, 10 mg Oxycodone/650 mg Acetaminophen	med	...	_
Friswell et al., 2008	9F/9M	Agonist	Morphine	PO	10 mg	low	...	_
		Agonist	Morphine	PO	10 mg	low	...	_
		Agonist	Oxycodone	PO	5 mg	low	...	_
		Agonist	Oxycodone	PO	5 mg	low	...	_
Quednow, Csomor, Chmiel, Beck, & Vollenweider, 2008	18M	Agonist	Morphine	PO	10 mg	high	...	_

F = female; M = male participants. Studies used within-subject designs unless otherwise noted.

^1^Estimated dose for agonists based on morphine equivalent of the maximum dose of in the respective study. For antagonist drugs, the estimated dose is preceded by a minus. See caption of Figure 2 for more details.

^2^Arrow indicates significantly improved (↑) , impaired (↓), mixed effects (↕) or null-effects (…) on performance relative to a placebo control condition.

^3^Minimum estimated dose at which the indicated effect is significantly different from placebo.

The handful of studies assessing effects of opioid agonist and antagonist treatment on mathematical skills are summarized in Table 4. The available evidence suggests that high doses of opioids drugs might impair several aspects of mathematical skills, including the speed at which participants complete oral and written addition tasks as reported by Smith and colleagues (Smith, Semke, & Beecher, 1962). However, other studies using low or moderate doses of opioid agonists failed to observe effects (Cleeland et al., 1996; Kornetsky, Humphries, & Evarts, 1957; Cherrier, Amory, Ersek, Risler, & Shen, 2009). Opioid blockade has not been shown to modulate arithmetic skills (Martín del Campo et al., 1992).

**Table 4:** Pharmacological studies reporting effect on mathematical skills

Reference	Sample	Drug type	Drug	Route	Dose	Max dose1	Effect2	Effect dose3
Kornetsky, Humphries, & Evarts, 1957	4F/6M	Agonist	Meperidine	PO	50, 100 mg	low	...	_
Smith, Semke, & Beecher, 1962 (Study 1)	24M	Agonist	Morphine	SC	10 mg	high	↓	high
Smith, Semke, & Beecher, 1962 (Study 2)	24M	Agonist	Heroin	SC	4 mg	high	↓	high
Smith, Semke, & Beecher, 1962 (Study 2)	24M	Agonist	Morphine	SC	10 mg	high	↓	high
Martín del Campo, McMurray, Besser, & Grossman, 1992	8M	Antagonist	Naloxone	IV	Cumulative infusion: 10-mg bolus was given =as a rapid bolus, followed by an infusion of 7 mg/hr for 12 hr	-med	...	_
Cleeland et al., 1996	5F/2M versus 2F/6M versus 5F/3M versus 3F/5M	Agonist	Morphine	PO	15, 20, 25, 30 mg	med	...	_

F = female; M = male participants. Studies used within-subject designs unless otherwise noted.

^1^Estimated dose for agonists based on morphine equivalent of the maximum dose of in the respective study. For antagonist drugs, the estimated dose is preceded by a minus. See caption of Figure 2 for more details.

^2^Arrow indicates significantly improved (↑) , impaired (↓), mixed effects (↕) or null-effects (…) on performance relative to a placebo control condition.

^3^Minimum estimated dose at which the indicated effect is significantly different from placebo.

Cognitive flexibility, another key aspect of executive function, is rarely investigated in the context of opioid drug studies. In an early study, Primac et al. (1957) did not find an effect of a low dose of the opioid agonist Meperidine administered to ten participants on the Wisconsin Card Sorting Test (WCST). More recently, Quednow and colleagues (Quednow, Csomor, Chmiel, Beck, & Vollenweider, 2008) using a low dose of 10 mg PO of morphine in 18 males did not observe effects on the Stockings of Cambridge tasks or extradimensional set switching. However, their low dose of morphine did reduce the error rate on intradimensional set shifts, suggesting that low doses of opioids might help to improve the application of a task rule within the same perceptual dimension.

The effects of opioid drugs on neuropsychological tests of executive function have most often been investigated in the domains of coding, logical reasoning, and working memory. While a large proportion of studies did observe opioid agonist-induced impairments in coding and logical reasoning, there are no consistent effects of opioid drugs on working memory.

### Attention

In an early study, Arnsten et al. (1983, 1984) hypothesized that blocking endogenous opioid activity might improve the selectivity of attention. Using a small dose (2 mg IV) of naloxone in an EEG study of ten male participants, they indeed observed that naloxone increased a late frontal event-related potential component, which is thought to reflect attention to auditory stimuli. Findings were consistent with their prior findings in animals (Arnsten et al., 1981) and were suggested to be driven by interactions with the locus-coeruleus-norepinephrine system. However, a more recent study testing four females and nine males using a higher dose of the opioid antagonist naltrexone (50 mg PO) to block >90% of mu-opioid receptors observed an effect in a direction opposite to the findings by Arnsten and colleagues (Jääskeläinen et al., 1998). These authors speculated that the observed impairment in selectivity of attention in their study might be due to nausea induced by naltrexone in their participants. Thus, a full opioid blockade (>90%) appears to cause the opposite attentional effect of weak opioid blockade. Other studies using high doses of opioid agonists did not observe clear impairments in divided attention task performance (Saarialho-Kere, 1988; Saarialho-Kere, Mattila, & Seppälä, 1989; for procedural details of these studies see Table 1). These null-effects were supported by the finding that a low dose of infused remifentanil (n = 14, within-subjects) impaired attention in a letter detection task only when participants expected that the drug would be administered but not when they did not expect the drug (open vs. hidden administration; Atlas, Wielgosz, Whittington, & Wager, 2014).

The ability to regulate sensory input by filtering out irrelevant stimuli (to prevent sensory overflow) can also be enhanced by opioid agonists, as suggested by a study where a low dose of morphine (10 mg PO) in 18 males enhanced modulation of the startle reflex to a noise burst following a prestimulus, a phenomenon called prepulse inhibition (Quednow et al., 2008). At first sight, these findings seem difficult to reconcile with the findings reported by Arnsten et al. (1983, 1984). As we will discuss in more detail later, one possibility is that the task by Quednow et al. (2008) involved increased levels of distress because of the loud auditory noise involved in the task. Opioids might help to downregulate stress responses under such conditions, perhaps improving sensorimotor gating relative to placebo. However, as reviewed elsewhere (Jacobson et al., 2018), effects of opioid drugs on prepulse inhibition in rodents are mixed, showing that additional systematic research is needed.

### Inhibition and effort

The effects of blocking opioid receptors on response inhibition were investigated by Martin del Campo et al. (1992) using the Stroop task and more recently in a Stroop-like prime-probe task by van Steenbergen et al. (2017). The first study used a cumulative infusion of naloxone in 8 males and the other administered 50 mg naltrexone PO versus placebo in two groups of 26 female participants. Both studies revealed that overall Stroop performance was not affected by the pharmacological manipulation. Likewise, no significant effects were reported by the earlier described study by Zacny and de Wit on impulsive action, which investigated the effect of 5, 10, and 20 mg PO of oxycodone in six females and six males participants on stop-signal performance and go/no-go performance (Zacny & de Wit, 2009). The absence of clear findings on overall measures of response inhibition resonates with rodent studies that often find no or mixed effects of opioid manipulations on premature responding (Jacobson et al., 2018).

The study by van Steenbergen and colleagues also analyzed post-error and post-conflict adjustments in behavioral performance (2017), which are thought to reflect short-term adaptive increases in cognitive control triggered by aversive arousal integral to the task at hand (Botvinick, Braver, Barch, Carter, & Cohen, 2001; Dreisbach & Fischer, 2012; Inzlicht et al., 2015; van Steenbergen, 2015). Naltrexone was observed to increase reaction time slowing after participants made an error. This finding suggests that the aversive arousal associated with conflict and errors can be increased when endogenous opioid activity is blocked, which improves short-term adaptive cognitive control. On the other hand, chronic stress and depression is associated with hyperactive neural error monitoring, impairing post-error accuracy (Pizzagalli, 2011). Recent work in rodents has shown that a kappa-specific antagonist can ameliorate stress-induced post-error impairments (Beard et al., 2015). This illustrates that modulation of aversive arousal is not restricted to mu-opioid receptors (Valentino & Van Bockstaele, 2015).

Two studies have investigated the effect of the opioid system on perceived task difficulty and required effort. Grossman et al. (1984) reported a study that tested the effect of an opioid blockade (12.2 mg IV of naloxone) in six male participants performing a physical exercise task. This opioid antagonist increased the perceived difficulty of the task. Two further studies reported that opioid blockade abolished exercise-induced mood improvements (Allen & Coen, 1987; Daniel, Martin, & Carter, 1992), conceivably through increased perceived difficulty. Interestingly, a more recent study has observed that the opioid agonist oxycodone (10 mg PO) administered to 18 participants did not affect driving performance, whereas they did report increases in required effort while performing the task (Verster, Veldhuijzen, & Volkerts, 2006). This finding points to the possibility that compensatory effort (Hockey, 1997) might mask opioid-related impairment in performance on cognitive control tasks.

### Integrative discussion of the reviewed literature

What can be learned from the budding literature on human opioid regulation of cognition and decision making? In light of the moderate to high density of mu-opioid receptors in the brain circuits involved in decision making and cognitive control (Figure 1), endogenous or drug modulation of mu-opioid receptors could exert direct modulatory effects on these processes. Nevertheless, considering all reviewed evidence together, one striking observation is the abundance of pharmacological studies observing null effects. This is true in particular for the delay-discounting tasks, working memory task and overall measures of planning, switching, and inhibition. However, the majority of these studies have used only small to moderate sample sizes, which usually are only adequately powered to observe medium to high effect sizes. Considering that typical effects sizes in the field of psychology and affective neuroscience are small to moderate (Lakens & Evers, 2014; Poldrack et al., 2017), the majority of the published studies were underpowered to detect such effects. One conclusion that can be drawn is thus that new research in this area must take measures to improve statistical power. In the meantime, we advise caution in the interpretation of these null effects, in particular if the study used low or moderate doses of opioid agonists only.

Null effects reported after full (>90%) blockade of mu-opioid receptors are an intermediate case, since valuable information can indeed be gleaned by observing behaviors unaltered or only partly diminished when opioid signaling is blocked. For instance, it is striking that healthy people display comparable working memory capacity and cognitive flexibility after treatment with opioid agonists, antagonists, and placebo. Similarly, although several studies report reduced reward pleasantness after naloxone or naltrexone, healthy humans consistently report substantial enjoyment of rewards when mu-opioid receptors are fully blocked. The clearest patterns indicating opioid modulation of performance emerged for value-based learning and decision-making tasks, the DSST, and the logical reasoning task. We will elaborate on these results below and highlight potential neural mechanisms.

### Reward-based decision-making

The reviewed evidence from studies of reward-based decision-making in humans is largely consistent with opioid regulation of reward motivation, as measured by effort invested to obtain relatively high-value rewards (Chelnokova et al., 2014; Eikemo et al., 2017; Weber et al., 2016). Extensive evidence from non-human animals indicates a parallel mechanism (Mahler & Berridge, 2012; S. Peciña & Berridge, 2013). We speculate that the rewarding effects of exerting physical effort during exercise (Allen & Coen, 1987; Daniel et al., 1992; Grossman et al., 1984; Hiura et al., 2017; Saanijoki et al., 2018) or cognitive effort (Inzlicht, Shenhav, & Olivola, 2018) may be mediated by the opioid system. Note that the existing literature in healthy humans does not allow us to disentangle the decision processes involved in weighing costs, such as effort expenditure, against gains, such as social, monetary, or taste rewards.

**Fig. 4:**
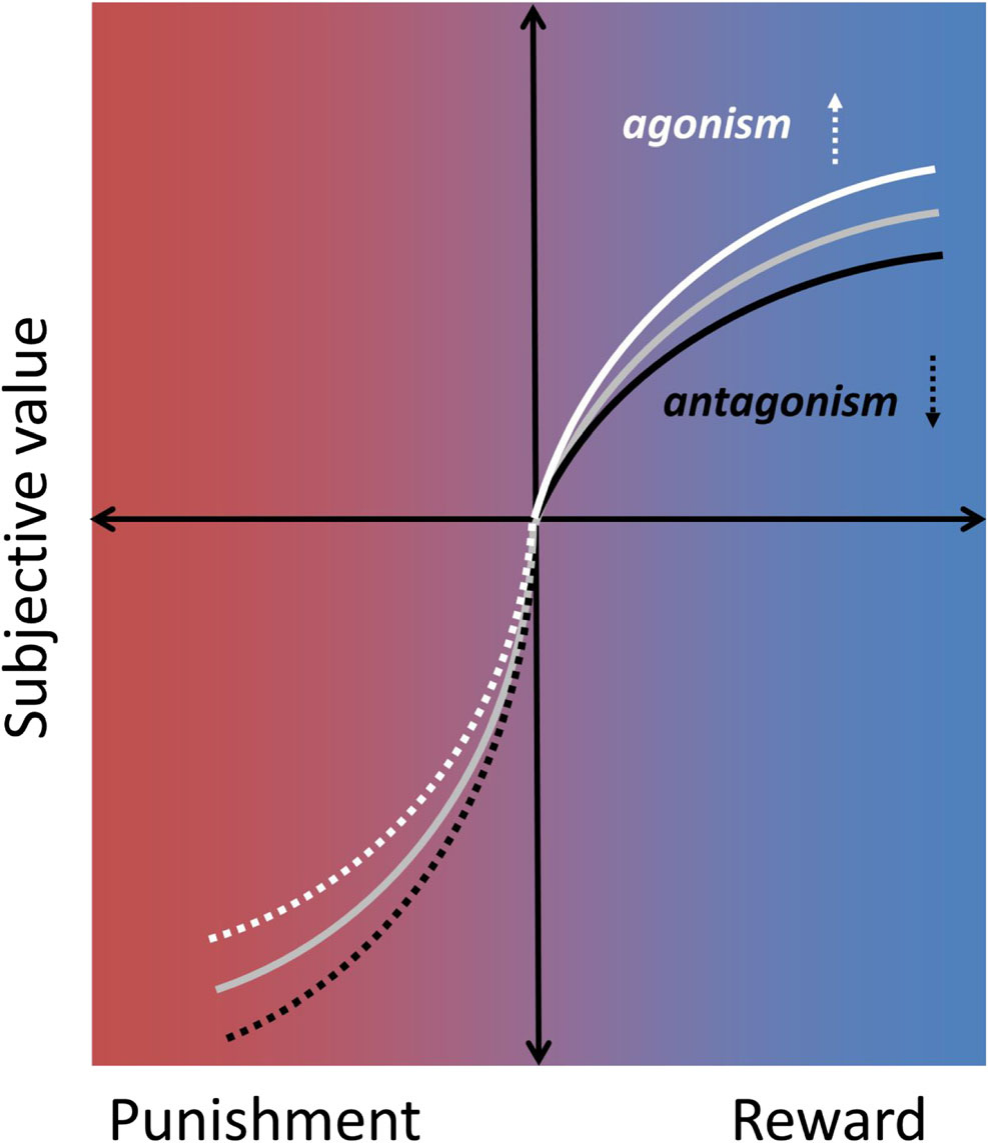
Subjective value as a function of reward and punishment. The gray line plots a typical value function for reward and punishment according to prospect theory (Kahneman & Tversky, 1979). Opioid drugs might modulate decision making by shifting this value function, such that opioid agonists increase (white line) and opioid antagonists decrease (black line) subjective value for rewards. Similar modulation specifically of high-salience stimuli may occur for punishments (dotted lines), although the available evidence is equivocal and future research is warranted (see *Discussion* in main text).

The current literature also points to a modest opioid modulation of the rewarding experience (liking) of high-value stimuli, but so far there is little evidence of a change in the neural response of winning money in healthy humans. One of the studies reviewed also points to opioid modulation of (monetary) reward learning (Efremidze et al., 2017). Overall, however, findings are in line with the notion that mu-opioid receptor stimulation by endogenous and exogenous opioid peptides causes a shift in valuation along a “hedonic gradient” ranging from unpleasant to pleasurable. As illustrated in Figure 4, we suggest that increased enjoyment of and motivation towards rewarding stimuli could underlie the observed changes in decision making. Studies in both rodents and humans indicate that these effects may be most pronounced for highly salient stimuli, such as high-value rewards.

Notably, these studies consistently show that blocking more than 90% of mu-opioid receptors does not obliviate the appreciation of a rewarded choice and only moderately reduces the pleasantness of rewards in general. Interestingly, while there is strong evidence that opioid drugs enhance food-liking responses in rodents, mu-opioid antagonism directly into “hedonic hotspots” did not suppress such appetite-independent “liking” (Smith & Berridge, 2007; Wassum et al., 2009b). However, systemic antagonism in rodents has been shown to suppress liking of sweet taste (Parker, Maier, Rennie, & Crebolder, 1992).

Opioid agonists might similarly attenuate the negative value of punishments, although the exact nature of this modulation requires extensive future research. For example, there is some evidence that opioid drugs may modulate large and small punishments to the same extent (Atlas et al., 2014; Gospic et al., 2007; Murray et al., 2014; Petrovic et al., 2008; Price, Harkins, Rafii, & Price, 1986; Schoell et al., 2010). Moreover, despite the relief opioid drugs can provide for acute clinical and experimental pain (Wanigasekera et al., 2012), psychosocial stress (Bershad et al., 2015, 2018), certain depressive symptoms (Peciña et al., 2018), and feelings of breathlessness (Hayen et al., 2017), opioid blockade does not consistently increase the aversiveness of experimental pain (Anderson et al., 2002; Berna et al., 2018; Eippert et al., 2008; Grevert & Goldstein, 1977). Clearly, more well-powered psychopharmacological evidence is needed to understand opioid modulation of reward and punishment processes, as well as their integration in the human brain.

### Cognitive control

With respect to the cognitive control domain, studies showed the most consistent effects (the highest proportion of significant effects) for the coding task (DSST). This is also the task that has been most frequently included in opioid drug studies. At moderate and high opioid drug doses, clear impairments on performance have been observed in many studies using this task (Figure 3). Although the DSST often is used as a primary measure of psychomotor skills, recent work using a factor-analytic approach suggest that performance on the DSST does not rely on basic psychomotor speed but instead relies on several executive function processes including working memory updating, switching, and inhibition (Knowles et al., 2015). This task might require a delicate coordination and integration of these different control processes. It could be speculated that this has rendered the DSST, and to a lesser extent logical reasoning, the most sensitive measures of cognitive control impairment. On the other hand, no evidence exists that blocking opioids enhances coding or logical reasoning performance in healthy people, which speaks against involvement of the endogenous opioid system as a key mechanism in executive functions. Indeed, other tasks in the cognitive domain, which are typically constructed to tap into a single subtype of control processes, were not associated with strong effects of opioid drugs or blockade. Combined, these results indicate that the effects of opioid drugs at moderate to high doses will be particularly strong for tasks which require the orchestration of multiple cognitive control functions relying on different regions in frontoparietal brain circuits. However, given the sparse data on cognitive measures other than DSST and logical reasoning, future research is warranted before firm conclusions can be drawn regarding the effects of opioids on these measures.

### Working hypothesis: Enhanced cognitive performance after opioid-reduced aversive arousal?

**Fig. 5:**
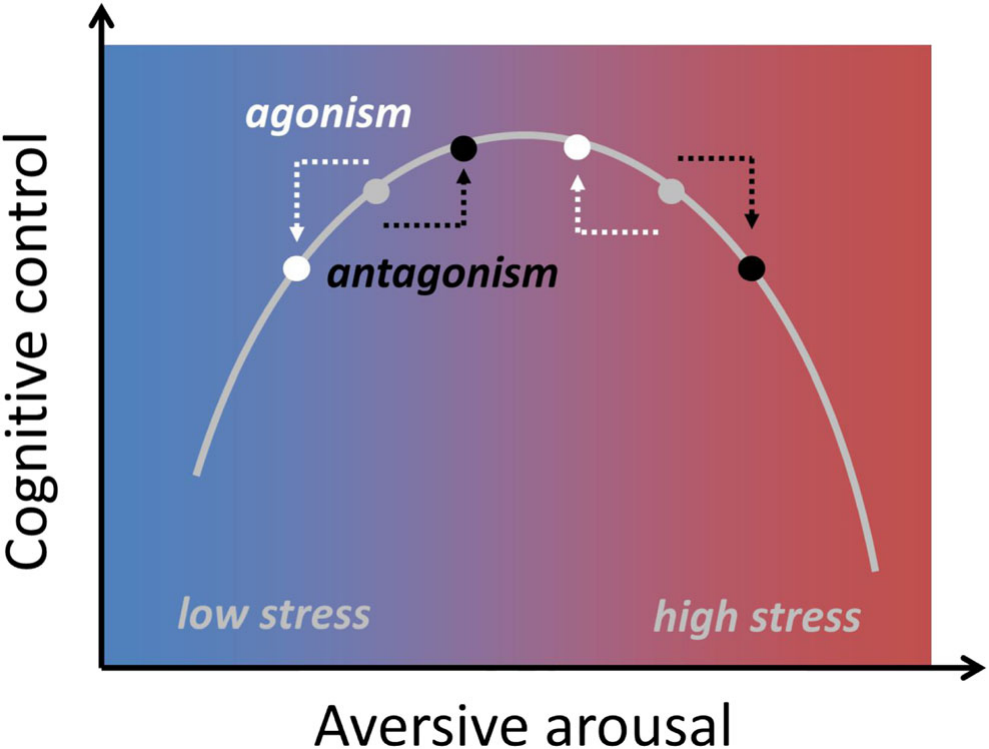
U-shaped relationship between aversive arousal and cognitive control. Opioids agonist might reduce aversive arousal (white), whereas opioid antagonist might increase it (black). According to this hypothesis, drug effects on cognition depend on the baseline level of aversive arousal, such that an opioid agonist might improve performance in high stress contexts, yet impair performance under low stress.

Some evidence suggests that opioid agonist administration relative to placebo can also improve rather than impair performance. This was for example observed for logical reasoning and DSST performance in early studies by Evans and colleagues (Evans & Smith, 1964; Evans & Witt, 1966) as well as in more recent work on intradimensional set shifting and attention (Quednow et al., 2008). These studies have in common that they used low doses of morphine (or codeine). One possible explanation for these findings might be that low doses of opioid agonists could reduce the aversive arousal (Thayer, 1989) or distress associated with a task. If the relationship between aversive arousal and cognitive control follows an inverted U-shape, as previous work has proposed (van Steenbergen, Band, & Hommel, 2015), low doses of opioid agonist drugs might compensate for arousal-induced impairment that occurs in the placebo condition (Figure 5). Opioids indeed tend to reduce arousal and can cause sedation at high doses. Even at lower doses, opioid agonists in humans and some other species reduce pupil size (miosis) (Lee & Wang, 1975; Murray, Adler, & Korczyn, 1983). In addition, opioid antagonism increases cortisol responses, and this is thought to reflect blockade of a tonic endogenous opioid inhibition of cortisol in humans (Lovallo et al., 2015).

The view that a low opioid drug dose could enhance cognitive performance by reducing aversive arousal also dovetails with recent rodent work on stress-alleviating properties of opioids (Valentino & Van Bockstaele, 2015). Endogenous opioid brain activation in response to stress might similarly help to prevent stress-induced impairments (Shields et al., 2016), as indeed suggested in some human (Bandura, Cioffi, Taylor, & Brouillard, 1988) and animal studies (Laredo et al., 2015). This view agrees with prior work that shows that cognitive control tasks elicit affective responses (i.e., integral emotions; Inzlicht et al., 2015), which might drive (mal) adaptive behavior (Botvinick, 2007; van Steenbergen et al., 2009) and which are likely under opioid regulation (van Steenbergen et al., 2017). Another possibility to consider is that cognitive improvements after low doses of opioids are caused by increases in appetitive motivation or learning. Such effects are particularly likely when the task itself involves external rewards (Eikemo et al., 2017) or when performance on tasks is perceived to reflect intelligence or capability. In addition, tasks perceived to be of high relevance might also generate internal “pseudo-rewards” (Holroyd & Yeung, 2012; Ribas-Fernandes et al., 2011), in particular when effort is intrinsically valuable (Inzlicht et al., 2018).

Given these considerations, it is plausible that cognitive control, just like decision-making, is modulated by opioids via brain networks involved in valuation, saliency, and motivation, shifting the cost-benefit trade-off, which in turn determines allocation of cognitive control (Shenhav, Botvinick, & Cohen, 2013). In addition, prefrontal networks involved in maintaining task-goal representations might be modulated directly via binding to its opioid receptors. In line with this suggestion, a recent PET study observed that high mu-opioid signaling (lower binding potential) in a ventral region of the lateral prefrontal cortex was positively related to performance on the Wisconsin Card Sorting Test in a group of patients with major depressive disorder (Light, Bieliauskas, & Zubieta, 2017). A possible mechanism at a neuronal level could be that stimulation of mu-opioid receptors suppresses interneuron spiking and increases glutamate-coded output of prefrontal neurons at multiple projection targets, which in turn might engender disorganized control and decision processes (Baldo, 2016).

## Directions for future research

The previous section provided some initial insights into the role of the mu-opioid system in higher-level cognitive function. Yet, numerous issues require future investigation. One unresolved challenge is that opioids might reduce cortical signaling without directly affecting performance in cognitive tasks, because participants use strategies to compensate for these deficits (Hockey, 1997). Future studies should include physiological measures, such as cardiovascular measures (Gendolla, Wright, & Richter, 2011; Kuipers et al., 2017; Spruit, Wilderjans, & van Steenbergen, 2018) and task-evoked pupil dilation (Kahneman, 1973; van der Wel & van Steenbergen, 2018) to investigate potential compensatory mechanisms. In addition, behavioral impairments in control tasks might reflect shifts in motivation instead of reflecting a cognitive incapability (Kurzban, Duckworth, Kable, & Myers, 2013; Shenhav et al., 2017). As we alluded to earlier, cognitive control shares many processes and brain circuits that also are important for value-based decision making, and future studies are warranted to understand the role of opioids in these processes (Berkman, Hutcherson, Livingston, Kahn, & Inzlicht, 2017).

Although opioid agonist drugs do not typically produce strong subjective effects at low doses (Hanks, O’Neill, Simpson, Wesnes, 1995), changes in self-reported mood and arousal are typically reported in many of the studies reviewed, most consistently at higher doses. The evidence for mood effects from opioid blockade on the other hand, is much less compelling (Berna et al., 2018; Eippert et al., 2008; Grevert & Goldstein, 1977). Interestingly, mood induction tasks appear to modulate endogenous opioid neurotransmission (Koepp et al., 2009; Prossin et al., 2015; Zubieta et al., 2003). One important avenue for future research is to understand the role of affective and motivational states in altered cognitive function. For example, studies might investigate whether variation in receptor binding potential or drug-induced changes in subjective state correlate with behavioral outcomes (Light et al., 2017; Weber et al., 2016). Researchers investigating the effect of hedonic states on cognitive control and decision making (Dreisbach & Goschke, 2004; Isen & Means, 1983; van Steenbergen, Band, & Hommel, 2010; Van Steenbergen, Band, Hommel, Rombouts, & Nieuwenhuis, 2015) and the influence of motivation on these processes (Botvinick & Braver, 2015; Braver et al., 2014; Pessoa, 2009) could use antagonist drugs to determine the role of endogenous mu-opioid neurotransmission in these effects. On a related note, more broadly defined control processes, such as mental flexibility and creativity, often have been related to positive affective states (Ashby, Isen, & Turken, 1999). It would be interesting to assess the role of the opioid system in these processes as well (Streufert & Gengo, 1993; Zacny, 1995).

Opioid administration at higher doses can produce other subjective effects that may influence task performance, such as nausea, difficulty concentrating, or sedation (Zacny & de Wit, 2009). Aversive side-effects could counteract a positive hedonic shift induced by opioid agonists or inflate the negative shift induced by opioid antagonism. Drowsiness and feeling spaced out from large opioid drug doses could also directly impact performance on cognitive and value-based tasks. Such confounds can be avoided by employing smaller drug doses (counteracting any loss in statistical power, e.g., by increasing sample sizes) or by employing active placebo treatments to ensure that drug conditions are matched on relevant side effects. In addition, future research could draw inspiration from anecdotal evidence that opioids can induce pain asymbolia, i.e., intact detection of pain but without the affective-motivational component (Berthier, Starkstein, & Leiguarda, 1988). Studies might therefore implement measures of motivation/detachment to measure the effects of opioid drugs on engagement with cognitive and decision-making tasks.

Another unresolved question relates to context and counterfactual outcomes. Do opioid drugs modulate high-value reward processes equally in the presence of punishments, such as pain or possible economic loss? As recently observed by Buchel et al. (2018), opioid blockade reduced pleasantness ratings of erotic stimuli significantly more than ratings of monetary wins. It is unclear whether the inclusion of highly salient erotic stimuli reduced the relative value of money during the experiment. As for tasks including punishments as well as rewards, it is possible that mu-opioid stimulation would cause a shift primarily of aversive stimuli but not rewards, because aversive stimuli are typically more salient (Kahneman & Tversky, 1979). Naloxone increased aversiveness of economic loss but not economic gain in Petrovic et al. (2008). Kut et al. (2011) found effects of naloxone on pain but not on pleasantness ratings of erotic stimuli. Also, two studies have reported decreased pleasantness of opioid drug effects during physical pain (Conley, Toledano, Apfelbaum, & Zacny, 1997; Zacny, McKay, et al., 1996b; but see Comer, Sullivan, Vosburg, Kowalczyk, & Houser, 2010). Studies including both positive and negative facial expressions provide mixed evidence, however, with opioid drug effects preferentially observed for negative or positive affective stimuli in different studies. (Bershad et al., 2016; Loseth et al., 2018; Syal et al., 2015; Wardle et al., 2016). Moreover, Berna et al. (2018) reported the largest naloxone reduction in pleasantness of the best possible (yet still painful) outcome, indicating that relative relief is opioid-dependent. Well-powered studies, including both rewarding and aversive outcomes, are needed to resolve these inconsistencies. In addition, opioid effects on value-based decision making should also be addressed during ongoing pain (Gandhi, Becker, & Schweinhardt, 2013) or other opioid-sensitive aversive states.

Although opioid drugs can exert direct effects on mu-opioid receptors expressed in the important hubs of the neural decision-making and cognitive-control networks (Figure 1), they can also act indirectly via other neurotransmitters. For example, the canonical disinhibition model of Johnson and North (1992) proposed that opioid drugs induce reward via increased dopamine signaling due to opioid inhibition of GABA interneurons in the ventral tegmental area. More recent work has shown that we are only at an early stage of understanding the exact role of dopamine signaling for opioid drug effects (Badiani, Belin, Epstein, Calu, & Shaham, 2011; Corre et al., 2018; Nutt, Lingford-Hughes, Erritzoe, & Stokes, 2015). For instance, mu-opioid receptor activation can have a net excitatory or net inhibitory effect on VTA neurons depending on a variety of pre- and postsynaptic mechanisms (Fields & Margolis, 2015). Furthermore, dopamine modulates cognition via different receptor types and pathways (Bromberg-Martin, Matsumoto, & Hikosaka, 2010; Cools, 2015), making direct comparisons difficult. The available evidence renders it unlikely that effects of opioid drugs can simply be explained in terms of dopaminergic modulation alone. Rodent findings that mu-opioids and dopamine play functionally different roles in the hedonic and motivational properties of reward (Berridge, 2007) need further examination in humans. For instance, some studies are beginning to manipulate opioids and dopamine pharmacologically using the same tasks (Porchet et al., 2013; Weber et al., 2016) or even combining an agonist for one system with an antagonist for the other (Jayaram-Lindström et al., 2017; Jayaram-Lindström, Wennberg, Hurd, & Franck, 2004; Roche et al., 2017).

The mu-opioid system also interacts with other neurotransmitters systems. For example, interactions between opioids and the locus-coeruleus-norepinephrine system are well-documented (Arnsten et al., 1981; Chaijale et al., 2013), and futures studies might investigate whether opioid drugs modulate cognitive processing via norepinephrine. There is also evidence that the endocannabinoid system interacts with opioid mechanisms that support reward (Rowland, Mukherjee, & Robertson, 2001; Solinas & Goldberg, 2005).

In addition to the literature reviewed, there is increasing evidence of opioid involvement in psychopathology. For example, chronic opioid antagonist administration may increase cognitive control and decrease the value of reinforcers in different patient groups (Lobmaier, Kunøe, Gossop, & Waal, 2011), and there is some evidence for dysregulation of mu-opioid receptor function across addictions (Ghitza et al., 2010; Mick et al., 2016). While chronic opioid misuse has been associated with maladaptive decisions (Giordano et al., 2002; Landes, Christensen, & Bickel, 2012), prolonged opioid blockade can reduce craving and drug taking in individuals with substance use disorder (Johnson, 2006; Mouaffak et al., 2017; Tanum et al., 2017). Interestingly, in addition to reducing craving and relapse in addiction, opioid blockade may reduce impulsive behaviors in kleptomania, pathological gambling, and “sex addiction” (Bostwick & Bucci, 2008; Grant, Kim, & Odlaug, 2009; Johnson, 2006; Lahti, Halme, Pankakoski, Sinclair, & Alho, 2010; Minozzi et al., 2011; Porchet et al., 2013; Rukstalis et al., 2005; Stotts, Dodrill, & Kosten, 2009). In a different line of research, recent work has suggested that opioid agonists might reduce depressive symptoms (Ehrich et al., 2015; Fava et al., 2016; Stanciu, Glass, & Penders, 2017; Yovell et al., 2016) and influence anhedonia (Barch, Pagliaccio, & Luking, 2015; Treadway, Bossaller, Shelton, & Zald, 2012), providing possible new avenues to treat patients with mood disorders.

## Conclusions

The present review supports a role for the opioid system in modulating some key aspects of cognitive control and decision-making. We have shown that the effects of reward-based decision-making by opioid drugs might be driven by a shift in valuation processes. At higher doses, opioid agonists can impair performance on neuropsychological executive function tasks involving coding and logical reasoning. At lower doses opioids can improve cognitive function, and the working hypothesis proposed suggests that these effects are driven by opioid-induced reduction of aversive arousal. We hope that this review provides an initial roadmap for future research to gain a better understanding of how opioids modulate cognition, affect, and their interactions.

## References

[CR1] Allen ME, Coen D (1987). Naloxone blooking of running-induced mood changes. Annals of Sports Medicine.

[CR2] Anderson WS, Sheth RN, Bencherif B, Frost JJ, Campbell JN (2002). Naloxone increases pain induced by topical capsaicin in healthy human volunteers. Pain.

[CR3] Arnsten AFT, Neville HJ, Hillyard SA, Janowsky DS, Salk T, Diego S (1984). Naloxone selective increases information measures processing in humans. Journal of Neuroscience.

[CR4] Arnsten AFT, Segal DS, Loughlin SE, Roberts DCS, Jolla L, Diego S (1981). Evidence for an interaction of opioid and noradrenergic locus coeruleus systems in the regulation of environmental stimulus-directed behavior. Brain Research.

[CR5] Arnsten AFT, Segal DS, Neville HJ, Hillyard SA, Janowsky DS, Judd LL, Bloom FE (1983). Naloxone augments electrophysiological signs of selective attention in man. Nature.

[CR6] Ashby FG, Isen AM, Turken AU (1999). A neuropsychological theory of positive affect and its influence on cognition a neuropsychological theory of positive affect and its influence on cognition. Psychological Review.

[CR7] Aston-Jones G, Cohen JD (2005). An integrative theory of locus coeruleus-norepinephrine function: Adaptive gain and optimal performance. Annual Review of Neuroscience.

[CR8] Atlas LY, Wielgosz J, Whittington RA, Wager TD (2014). Specifying the non-specific factors underlying opioid analgesia: Expectancy, attention, and affect. Psychopharmacology.

[CR9] Badiani A, Belin D, Epstein D, Calu D, Shaham Y (2011). Opiate versus psychostimulant addiction: the differences do matter. Nature Reviews Neuroscience.

[CR10] Baldo BA (2016). Prefrontal cortical opioids and dysregulated motivation: A network hypothesis. Trends in Neurosciences.

[CR11] Bandura A, Cioffi D, Taylor CB, Brouillard ME (1988). Perceived self-efficacy in coping with cognitive stressors and opioid activation. Journal of Personality and Social Psychology.

[CR12] Barbano MF, Cador M (2007). Opioids for hedonic experience and dopamine to get ready for it. Psychopharmacology.

[CR13] Barch, D. M., Pagliaccio, D., & Luking, K. (2015). Mechanisms underlying motivational deficits in psychopathology: similarities and differences in depression and schizophrenia. In *Behavioral neuroscience of motivation* (pp. 411–449). Springer. 10.1007/7854_2015_376 26026289

[CR14] Bartra O, McGuire JT, Kable JW (2013). The valuation system: A coordinate-based meta-analysis of BOLD fMRI experiments examining neural correlates of subjective value. NeuroImage.

[CR15] Beard C, Donahue RJ, Dillon DG, Van’t Veer A, Webber C, Lee J (2015). Abnormal error processing in depressive states: a translational examination in humans and rats. Translational Psychiatry.

[CR16] Berkman, E. T., Hutcherson, C. A., Livingston, J. L., Kahn, L. E., & Inzlicht, M. (2017). Self-control as value-based choice. *Current Directions in Psychological Science, 26,* 422–428 . 10.1177/0963721417704394 PMC5765996 29335665

[CR17] Berna C, Leknes S, Ahmad AH, Mhuircheartaigh RN, Goodwin GM, Tracey I (2018). Opioid-independent and opioid-mediated modes of pain modulation. Journal of Neuroscience.

[CR18] Berridge KC (2007). The debate over dopamine’s role in reward: the case for incentive salience. Psychopharmacology.

[CR19] Berridge KC, Kringelbach ML (2015). Pleasure systems in the brain. Neuron.

[CR20] Berridge KC, Robinson TE, Aldridge JW (2009). Dissecting components of reward: “liking”, “wanting”, and learning. Current Opinion in Pharmacology.

[CR21] Bershad AK, Jaffe JH, Childs E, de Wit H (2015). Opioid partial agonist buprenorphine dampens responses to psychosocial stress in humans. Psychoneuroendocrinology.

[CR22] Bershad AK, Miller MA, Norman GJ, de Wit H (2018). Effects of opioid-and non-opioid analgesics on responses to psychosocial stress in humans. Hormones and Behavior.

[CR23] Bershad AK, Seiden JA, de Wit H (2016). Effects of buprenorphine on responses to social stimuli in healthy adults. Psychoneuroendocrinology.

[CR24] Berthier M, Starkstein S, Leiguarda R (1988). Asymbolia for pain: A sensory-limbic disconnection syndrome. Annals of Neurology.

[CR25] Bickel WK, Yi R, Landes RD, Hill PF, Baxter C (2011). Remember the future: Working memory training decreases delay discounting among stimulant addicts. Biological Psychiatry.

[CR26] Black ML, Hill JL, Zacny JP (1999). Behavioral and physiological effects of remifentanil and alfentanil in healthy volunteers. Anesthesiology.

[CR27] Boettiger CA, Kelley EA, Mitchell JM, D’Esposito M, Fields HL (2009). Now or later? An fMRI study of the effects of endogenous opioid blockade on a decision-making network. Pharmacology Biochemistry and Behavior.

[CR28] Bostwick JM, Bucci JA (2008). Internet sex addiction treated with naltrexone. Mayo Clinic Proceedings.

[CR29] Botvinick MM (2007). Conflict monitoring and decision making: Reconciling two perspectives on anterior cingulate function. Cognitive Affective & Behavioral Neuroscience.

[CR30] Botvinick MM, Braver TS (2015). Motivation and cognitive control: From behavior to neural mechanism. Annual Review of Psychology.

[CR31] Botvinick MM, Braver TS, Barch DMM, Carter CSS, Cohen JDD (2001). Conflict monitoring and cognitive control. Psychological Review.

[CR32] Bradley CM, Nicholson AN (1986). Effects of a mu-opioid receptor agonist (codeine phosphate) on visuo-motor coordination and dynamic visual acuity in man. British Journal of Clinical Pharmacology.

[CR33] Bradley CM, Nicholson AN (1987). Studies on performance with aspirin and paracetamol and with the centrally acting analgesics meptazinol and pentazocine. European Journal of Clinical Pharmacology.

[CR34] Braver, T. S., Krug, M. K., Chiew, K. S., Kool, W., Westbrook, J. A., Clement, N. J., … Somerville, L. H. (2014). Mechanisms of motivation-cognition interaction: challenges and opportunities. *Cognitive, Affective & Behavioral Neuroscience*, 443–472. 10.3758/s13415-014-0300-0 PMC4986920 24920442

[CR35] Bromberg-Martin ES, Matsumoto M, Hikosaka O (2010). Dopamine in motivational control: Rewarding, aversive, and alerting. Neuron.

[CR36] Buchel, C., Miedl, S., & Sprenger, C. (2018). Hedonic processing in humans is mediated by an opioidergic mechanism in a mesocorticolimbic system. *ELife*, 7:e39648. 10.7554/eLife.39648 PMC6239433 30444488

[CR37] Calo G, Guerrini R, Rizzi A, Salvadori S, Regoli D (2000). Pharmacology of nociceptin and its receptor: a novel therapeutic target. British Journal of Pharmacology.

[CR38] Castro DC, Berridge KC (2017). Opioid and orexin hedonic hotspots in rat orbitofrontal cortex and insula. Proceedings of the National Academy of Sciences.

[CR39] Chaijale NN, Curtis AL, Wood SK, Zhang XY, Bhatnagar S, Reyes BA (2013). Social stress engages opioid regulation of locus coeruleus norepinephrine neurons and induces a state of cellular and physical opiate dependence. Neuropsychopharmacology.

[CR40] Chelnokova O, Laeng B, Eikemo M, Riegels J, Løseth G, Maurud H (2014). Rewards of beauty: the opioid system mediates social motivation in humans. Molecular Psychiatry.

[CR41] Chelnokova, O., Laeng, B., Løseth, G., Eikemo, M., Willoch, F., & Leknes, S. (2016). The μ-opioid system promotes visual attention to faces and eyes. *Social Cognitive and Affective Neuroscience, 11,* 1902–1909. 10.1093/scan/nsw116 PMC5141966 27531386

[CR42] Cherrier MM, Amory JK, Ersek M, Risler L, Shen DD (2009). Comparative cognitive and subjective side effects of immediate-release oxycodone in healthy middle-aged and older adults. Journal of Pain.

[CR43] Chiew KS, Braver TS (2011). Positive affect versus reward: emotional and motivational influences on cognitive control. Frontiers in Psychology.

[CR44] Cleeland CS, Nakamura Y, Howland EW, Morgan NR, Edwards KR, Backonja M (1996). Effects of oral morphine on cold pressor tolerance time and neuropsychological performance. Neuropsychopharmacology.

[CR45] Cohen, R. M., Murphy, D. L., Cohen, R., Weingartner, H., & Pickar, D. (1983). High-dose naloxone affects task performance in normal subject, *36*, 127–136. 10.1016/0165-1781(83)90100-2 6304800

[CR46] Comer SD, Sullivan MA, Vosburg SK, Kowalczyk WJ, Houser J (2010). Abuse liability of oxycodone as a function of pain and drug use history. Drug and Alcohol Dependence.

[CR47] Conley KM, Toledano AY, Apfelbaum JL, Zacny JP (1997). Modulating effects of a cold water stimulus on opioid effects in volunteers. Psychopharmacology (Berl).

[CR48] Cools R (2015). The cost of dopamine for dynamic cognitive control. Current Opinion in Behavioral Sciences.

[CR49] Corbett AD (2009). 75 Years of opioid research: The exciting but vain quest for the Holy Grail. British Journal of Pharmacology.

[CR50] Corre J, van Zessen R, Loureiro M, Patriarchi T, Tian L, Pascoli V, Lüscher C (2018). Dopamine neurons projecting to medial shell of the nucleus accumbens drive heroin reinforcement. Elife.

[CR51] Costa PT, McCrae RR (1992). Normal personality assessment in clinical practice: The NEO Personality Inventory. Psychological Assessment.

[CR52] Daniel M, Martin AD, Carter J (1992). Opiate receptor blockade by naltrexone and mood state after acute physical activity. British Journal of Sports Medicine.

[CR53] De Quincey, T. (2000). *Confessions of an English Opium-eater*. *The Works of Thomas De Quincey, Vol. 2: Confessions of an English Opium-Eater, 1821–1856* (Vol. 89). Oxford University Press.

[CR54] Dreisbach G, Fischer R (2012). Conflicts as aversive signals. Brain and Cognition.

[CR55] Dreisbach G, Fischer R (2015). Conflicts as aversive signals for control adaptation. Current Directions in Psychological Science.

[CR56] Dreisbach G, Goschke T (2004). How positive affect modulates cognitive control: Reduced perseveration at the cost of increased distractibility. Journal of Experimental Psychology-Learning Memory and Cognition.

[CR57] Drewnowski A, Krahn DD, Demitrack MA, Nairn K, Gosnell BA (1992). Taste responses and preferences for sweet high-fat foods: Evidence for opioid involvement. Physiology and Behavior.

[CR58] Efremidze L, Sarraf G, Miotto K, Zak PJ (2017). The neural inhibition of learning increases asset market bubbles: Experimental evidence. Journal of Behavioral Finance.

[CR59] Ehrich E, Turncliff R, Du Y, Leigh-Pemberton R, Fernandez E, Jones R, Fava M (2015). Evaluation of opioid modulation in major depressive disorder. Neuropsychopharmacology.

[CR60] Eikemo M, Biele G, Willoch F, Thomsen L, Leknes S (2017). Opioid modulation of value-based decision-making in healthy humans. Neuropsychopharmacology.

[CR61] Eikemo, M., Løseth, G. E., Johnstone, T., Gjerstad, J., Willoch, F., & Leknes, S. (2016). Sweet taste pleasantness is modulated by morphine and naltrexone. *Psychopharmacology*, 1–13. 10.1007/s00213-016-4403-x 27538675

[CR62] Eippert F, Bingel U, Schoell E, Yacubian J, Büchel C (2008). Blockade of endogenous opioid neurotransmission enhances acquisition of conditioned fear in humans. Journal of Neuroscience.

[CR63] Ersek M, Cherrier MM, Overman SS, Irving GA (2004). The cognitive effects of opioids. Pain Management Nursing.

[CR64] Escher M, Daali Y, Chabert J, Hopfgartner G, Dayer P, Desmeules J, J EMYJGP (2007). Pharmacokinetic and pharmacodynamic properties of buprenorphine after a single intravenous administration in healthy volunteers: a randomized, double-blind, placebo-controlled, crossover study. Clinical Therapeutics.

[CR65] Evans WO, Smith RP (1964). Some effects of morphine and amphetamine on intellectual functions and mood. Psychopharmacologia.

[CR66] Evans WO, Witt NF (1966). The interaction of high altitude and psychotropic drug action. Psychopharmacologia.

[CR67] Fava M, Memisoglu A, Thase ME, Bodkin JA, Trivedi MH, De Somer M, Ehrich E (2016). Opioid modulation with buprenorphine/samidorphan as adjunctive treatment for inadequate response to antidepressants: A randomized double-blind placebo-controlled trial. American Journal of Psychiatry.

[CR68] Fichna J, Janecka A, Costentin J, Do Rego J-C (2007). The endomorphin system and its evolving neurophysiological role. Pharmacological Reviews.

[CR69] Fields HL, Margolis EB (2015). Understanding opioid reward. Trends in Neurosciences.

[CR70] File SE, Silverstone T (1981). Naloxone changes self-ratings but not performance in normal subjects. Psychopharmacology.

[CR71] Friswell J, Phillips C, Holding J, Morgan CJA, Brandner B, Curran HV (2008). Acute effects of opioids on memory functions of healthy men and women. Psychopharmacology.

[CR72] Gandhi W, Becker S, Schweinhardt P (2013). Pain increases motivational drive to obtain reward, but does not affect associated hedonic responses: A behavioural study in healthy volunteers. European Journal of Pain.

[CR73] Gendolla GHE, Wright RA, Richter M, Ryan R (2011). Effort intensity: Studies of cardiovascular response. The Oxford handbook on motivation.

[CR74] Ghitza UE, Preston KL, Epstein DH, Kuwabara H, Endres CJ, Bencherif B (2010). Brain mu-opioid receptor binding predicts treatment outcome in cocaine-abusing outpatients. Biological Psychiatry.

[CR75] Ghoneim MM, Mewaldt SP, Thatcher JW (1975). The effect of diazepam and fentanyl on mental, psychomotor and electroencephalographic functions and their rate of recovery. Psychopharmacologia.

[CR76] Giordano L, Bickel W, Loewenstein G, Jacobs E, Marsch L, Badger G (2002). Mild opioid deprivation increases the degree that opioid-dependent outpatients discount delayed heroin and money. Psychopharmacology.

[CR77] Gorgolewski KJ, Varoquaux G, Rivera G, Schwarz Y, Ghosh SS, Maumet C (2015). NeuroVault.org: A web-based repository for collecting and sharing unthresholded statistical maps of the human brain. Frontiers in Neuroinformatics.

[CR78] Gospic K, Gunnarsson T, Fransson P, Ingvar M, Lindefors N, Petrovic P (2007). Emotional perception modulated by an opioid and a cholecystokinin agonist. Psychopharmacology.

[CR79] Grant JE, Kim SW, Odlaug BL (2009). A double-blind, placebo-controlled study of the opiate antagonist, naltrexone, in the treatment of kleptomania. Biological Psychiatry.

[CR80] Grevert P, Goldstein A (1977). Effects of naloxone on experimentally induced ischemic pain and on mood in human subjects. Proceedings of the National Academy of Sciences.

[CR81] Grossman A, Bouloux P, Price P, Drury PL, Lam KSL, Turner T, Sutton J (1984). The role of opioid peptides in the hormonal responses to acute exercise in man. Clinical Science.

[CR82] Haaker J, Yi J, Petrovic P, Olsson A (2017). Endogenous opioids regulate social threat learning in humans. Nature Communications.

[CR83] Hanks, G., O’Neill, W., Simpson, P., Wesnes, K., & A, I. (1995). The cognitive and psychomotor effects of opioid analgesics. II. A randomized controlled trial of single doses of morphine, lorazepam and placebo in healthy subjects. *European Journal of Clinical Pharmacology*, *48*, 455–460. 10.1007/BF00194334 8582463

[CR84] Hayen A, Wanigasekera V, Faull OK, Campbell SF, Garry PS, Raby SJM (2017). Opioid suppression of conditioned anticipatory brain responses to breathlessness. Neuroimage.

[CR85] Henriksen G, Willoch F (2008). Imaging of opioid receptors in the central nervous system. Brain.

[CR86] Hill JL, Zacny JP (2000). Comparing the subjective, psychomotor, physiological effects of intravenous hydromorphone and morphine healthy volunteers. Psychopharmacology.

[CR87] Hiura M, Sakata M, Ishii K, Toyohara J, Oda K, Nariai T, Ishiwata K (2017). Central μ-opioidergic system activation evoked by heavy and severe-intensity cycling exercise in humans: a pilot study using positron emission tomography with 11C-Carfentanil. International Journal of Sports Medicine.

[CR88] Hockey GRJRJ (1997). Compensatory control in the regulation of human performance under stress and high workload: A cognitive-energetical framework. Biological Psychology.

[CR89] Holroyd CB, Yeung N (2012). Motivation of extended behaviors by anterior cingulate cortex. Trends in Cognitive Sciences.

[CR90] Hsu DT, Sanford BJ, Meyers KK, Love TM, Hazlett KE, Wang H (2013). Response of the μ-opioid system to social rejection and acceptance. Molecular Psychiatry.

[CR91] Inzlicht M, Bartholow BD, Hirsh JB (2015). Emotional foundations of cognitive control. Trends in Cognitive Sciences.

[CR92] Inzlicht, M., Shenhav, A., & Olivola, C. Y. (2018). The effort paradox: Effort is both costly and valued. *Trends in Cognitive Sciences, 22,* 337–349. 10.1016/j.tics.2018.01.007 PMC6172040 29477776

[CR93] Isen AM, Means B (1983). The influence of positive affect on decision-making strategy. Social Cognition.

[CR94] Jääskeläinen IP, Hirvonen J, Kujala T, Alho K, Eriksson CJP, Lehtokoski A, Sillanaukee P (1998). Effects of naltrexone and ethanol on auditory event-related brain potentials. Alcohol.

[CR95] Jacob JJC, Michaud GM, Tremblay EC (1979). Mixed agonist-antagonist opiates and physical dependence. British Journal of Clinical Pharmacology.

[CR96] Jacobson ML, Wulf HA, Browne CA, Lucki I, O’Mara S (2018). Opioid modulation of cognitive impairment in depression. Progress in brain research.

[CR97] Jarvik LF, Simpson JH, Guthrie D, Liston EH (1981). Morphine, experimental pain, and psychological reactions. Psychopharmacology.

[CR98] Jayaram-Lindström N, Guterstam J, Häggkvist J, Ericson M, Malmlöf T, Schilström B, Franck J (2017). Naltrexone modulates dopamine release following chronic, but not acute amphetamine administration: a translational study. Translational Psychiatry.

[CR99] Jayaram-Lindström N, Wennberg P, Hurd YL, Franck J (2004). Effects of naltrexone on the subjective response to amphetamine in healthy volunteers. Journal of Clinical Psychopharmacology.

[CR100] Johnson BA (2006). A synopsis of the pharmacological rationale, properties and therapeutic effects of depot preparations of naltrexone for treating alcohol dependence. Expert Opinion on Pharmacotherapy.

[CR101] Johnson SW, North RA (1992). Opioids excite dopamine neurons by hyperpolarization of local interneurons. Journal of Neuroscience.

[CR102] Kahneman D (1973). Attention and effort.

[CR103] Kahneman D, Tversky A (1979). Prospect theory: An analysis of decisions under risk. Econometrica.

[CR104] Kieres AK, Hausknecht KA, Farrar AM, Acheson A, de Wit H, Richards JB (2004). Effects of morphine and naltrexone on impulsive decision making in rats. Psychopharmacology.

[CR105] Knotkova H, Fine PG, Portenoy RK (2009). The puzzle of processing speed, memory, and executive function impairments in schizophrenia: Fitting the pieces together. Journal of Pain and Symptom Management.

[CR106] Knowles EEM, Weiser M, David AS, Glahn DC, Davidson M, Reichenberg A (2015). The Puzzle of Processing Speed, Memory, and Executive Function Impairments in Schizophrenia: Fitting the Pieces Together. Biological Psychiatry.

[CR107] Koepp MJ, Hammers A, Lawrence AD, Asselin MC, Grasby PM, Bench CJ (2009). Evidence for endogenous opioid release in the amygdala during positive emotion. NeuroImage.

[CR108] Koob GF, Le Moal M (2001). Drug addiction, dysregulation of reward, and allostasis. Neuropsychopharmacology.

[CR109] Kornetsky C, Humphries O, Evarts EV (1957). Comparison of Psychological Effects of Certain Centrally Acting Drugs in Man. Archives of Neurology And Psychiatry.

[CR110] Kringelbach ML, Berridge KC (2009). Towards a functional neuroanatomy of pleasure and happiness. Trends in Cognitive Sciences.

[CR111] Kuipers, M., Richter, M., Scheepers, D., Immink, M. A., Sjak-Shie, E., & van Steenbergen, H. (2017). How effortful is cognitive control? Insights from a novel method measuring single-trial evoked beta-adrenergic cardiac reactivity. *International Journal of Psychophysiology*, *119,* 87–92. 10.1016/j.ijpsycho.2016.10.007 27737782

[CR112] Kurzban R, Duckworth A, Kable JW, Myers J (2013). An opportunity cost model of subjective effort and task performance. Behavioral and Brain Sciences.

[CR113] Kut E, Candia V, von Overbeck J, Pok J, Fink D, Folkers G (2011). Pleasure-Related Analgesia Activates Opioid-Insensitive Circuits. Journal of Neuroscience.

[CR114] Lahti T, Halme JT, Pankakoski M, Sinclair D, Alho H (2010). Treatment of pathological gambling with naltrexone pharmacotherapy and brief intervention: A pilot study. Psychopharmacology Bulletin.

[CR115] Lakens D, Evers ERK (2014). Sailing from the seas of chaos into the corridor of stability: Practical recommendations to increase the informational value of studies. Perspectives on Psychological Science.

[CR116] Landes RD, Christensen DR, Bickel WK (2012). Delay discounting decreases in those completing treatment for opioid dependence. Experimental and Clinical Psychopharmacology.

[CR117] Laredo SA, Steinman MQ, Robles CF, Ferrer E, Ragen BJ, Trainor BC (2015). Effects of defeat stress on behavioral flexibility in males and females: Modulation by the mu-opioid receptor. European Journal of Neuroscience.

[CR118] Laurent V, Morse AK, Balleine BW (2015). The role of opioid processes in reward and decision-making. British Journal of Pharmacology.

[CR119] Lee HK, Wang SC (1975). Mechanism of morphine-induced miosis in the dog. The Journal of Pharmacology and Experimental Therapeutics.

[CR120] Leknes S, Tracey I (2008). A common neurobiology for pain and pleasure. Nature Reviews Neuroscience.

[CR121] Light SN, Bieliauskas LA, Zubieta J-K (2017). “Top-down” mu-opioid system function in humans: Mu-opioid receptors in ventrolateral prefrontal cortex mediate the relationship between hedonic tone and executive function in major depressive disorder. The Journal of Neuropsychiatry and Clinical Neurosciences.

[CR122] Lobmaier PP, Kunøe N, Gossop M, Waal H (2011). Naltrexone depot formulations for opioid and alcohol dependence: A systematic review. CNS Neuroscience and Therapeutics.

[CR123] Loseth GE, Eikemo M, Isager P, Holmgren J, Laeng B, Vindenes V, Hjørnevik T (2018). Morphine reduced perceived anger from neutral and implicit emotional expressions. Psychoneuroendocrinology.

[CR124] Loseth GE, Ellingsen D-M, Leknes S (2014). State-dependent mu-opioid modulation of social motivation. Frontiers in Behavioral Neuroscience.

[CR125] Lovallo WR, Enoch MA, Acheson A, Cohoon AJ, Sorocco KH, Hodgkinson CA, Goldman D (2015). Cortisol stress response in men and women modulated differentially by the mu-opioid receptor gene polymorphism OPRM1 A118G. Neuropsychopharmacology.

[CR126] Love TM, Stohler CS, Zubieta J-K (2009). Positron emission tomography measures of endogenous opioid neurotransmission and impulsiveness traits in humans. Archives of General Psychiatry.

[CR127] Lutz PE, Kieffer BL (2013). The multiple facets of opioid receptor function: implications for addiction. Current Opinion in Neurobiology.

[CR128] MacDonald F, Gough K, Nicoll R, Dow R (1989). Psychomotor effects of ketorolac in comparison with buprenorphine and diclofenac. British Journal of Clinical Pharmacology.

[CR129] Mahler SV, Berridge KC (2012). What and when to “want”? Amygdala-based focusing of incentive salience upon sugar and sex. Psychopharmacology.

[CR130] Marsch LA, Bickel WK, Badger GJ, Rathmell JP, Swedberg MDB, Jonzon B, Norsten-Hoog C (2001). Effects of infusion rate of intravenously administered morphine on physiological, psychomotor, and self-reported measures in humans. Journal of Pharmacology and Experimental Therapeutics.

[CR131] Martín del Campo AF, McMurray RG, Besser GM, Grossman A (1992). Effect of 12-hour infusion of naloxone on mood and cognition in normal male volunteers. Biological Psychiatry.

[CR132] Mayberg, H. S., & Frost, J. J. (1990). Opiate receptors. In *Quantitative imaging: neuroreceptors, neurotransmitters, and enzymes* (pp. 81–95). New York: Raven Press.

[CR133] Mick I, Myers J, Ramos AC, Stokes PRA, Erritzoe D, Colasanti A (2016). Blunted endogenous opioid release following an oral amphetamine challenge in pathological gamblers. Neuropsychopharmacology.

[CR134] Minozzi, S., Amato, L., Vecchi, S., Davoli, M., Kirchmayer, U., & Verster, A. (2011). Oral naltrexone maintenance treatment for opioid dependence. *Cochrane Database Syst Rev*, CD001333. 10.1002/14651858.CD001333.pub2 16437431

[CR135] Mitchell JM, Tavares VC, Fields HL, D’Esposito M, Boettiger CA (2007). Endogenous opioid blockade and impulsive responding in alcoholics and healthy controls. Neuropsychopharmacology.

[CR136] Mouaffak F, Leite C, Hamzaoui S, Benyamina A, Laqueille X, Kebir O (2017). Naltrexone in the treatment of broadly defined behavioral addictions: A review and meta-analysis of randomized controlled trials. European Addiction Research.

[CR137] Mucha RF, Iversen SD (1984). Reinforcing properties of morphine and naloxone revealed by conditioned place preferences: a procedural examination. Psychopharmacology.

[CR138] Murray E, Brouwer S, McCutcheon R, Harmer CJ, Cowen PJ, McCabe C (2014). Opposing neural effects of naltrexone on food reward and aversion: implications for the treatment of obesity. Psychopharmacology.

[CR139] Murray, R. B., Adler, M. W., & Korczyn, A. D. (1983). The pupillary effects of oploids. *Life Sciences*. 10.1016/0024-3205(83)90123-6 6136886

[CR140] Nestor LJ, Murphy A, McGonigle J, Orban C, Reed L, Taylor E (2017). Acute naltrexone does not remediate fronto-striatal disturbances in alcoholic and alcoholic polysubstance-dependent populations during a monetary incentive delay task. Addiction Biology.

[CR141] Notebaert W, Braem S, Braver TS (2016). Parsing the effect of reward on cognitive control. Motivation and cognitive control.

[CR142] Nummenmaa L, Saanijoki T, Tuominen L, Hirvonen J, Tuulari JJ, Nuutila P, Kalliokoski K (2018). Mu-opioid receptor system mediates reward processing in humans. Nature Communications.

[CR143] Nummenmaa, L., & Tuominen, L. (2018). Opioid system and human emotions. *British Journal of Pharmacology, 175,* 2737–2749. 10.1111/bph.13812 PMC6016642 28394427

[CR144] Nutt DJ, Lingford-Hughes A, Erritzoe D, Stokes PR (2015). The dopamine theory of addiction: 40 years of highs and lows. Nat Rev Neurosci.

[CR145] Oliveto AH, Bickel WK, Kamien JB, Hughes JR, Higgins ST (1994). Effects of diazepam and hydromorphone in triazolam-trained humans under a novel-response drug discrimination procedure. Psychopharmacology.

[CR146] Parker LA, Maier S, Rennie M, Crebolder J (1992). Morphine- and naltrexone-induced modification of palatability: Analysis by the taste reactivity test. Behavioral Neuroscience.

[CR147] Pasternak GW (2001). Insights into mu opioid pharmacology: The role of mu opioid receptor subtypes. Life Sciences.

[CR148] Pattij T, Schetters D, Janssen MCW, Wiskerke J, Schoffelmeer ANM (2009). Acute effects of morphine on distinct forms of impulsive behavior in rats. Psychopharmacology.

[CR149] Peciña, M., Karp, J. F., Mathew, S., Todtenkopf, M. S., Ehrich, E. W., & Zubieta, J. K. (2019). Endogenous opioid system dysregulation in depression: implications for new therapeutic approaches. *Molecular Psychiatry, 24*, 576–587. 10.1038/s41380-018-0117-2 PMC6310672 29955162

[CR150] Peciña S, Berridge KC (2013). Dopamine or opioid stimulation of nucleus accumbens similarly amplify cue-triggered “wanting” for reward: Entire core and medial shell mapped as substrates for PIT enhancement. European Journal of Neuroscience.

[CR151] Peciña S, Smith KS (2010). Hedonic and motivational roles of opioids in food reward: Implications for overeating disorders. Pharmacology Biochemistry and Behavior.

[CR152] Pessoa L (2009). How do emotion and motivation direct executive control?. Trends in Cognitive Sciences.

[CR153] Petrovic P, Pleger B, Seymour B, Kloppel S, De Martino B, Critchley H, Dolan RJ (2008). Blocking central opiate function modulates hedonic impact and anterior cingulate response to rewards and losses. Journal of Neuroscience.

[CR154] Pizzagalli DA (2011). Frontocingulate dysfunction in depression: Toward biomarkers of treatment response. Neuropsychopharmacology.

[CR155] Poldrack RA, Baker CI, Durnez J, Gorgolewski KJ, Matthews PM, Munafò MR (2017). Scanning the horizon: Towards transparent and reproducible neuroimaging research. Nature Reviews Neuroscience.

[CR156] Porchet, R., Boekhoudt, L., Studer, B., Gandamaneni, K., Rani, N., Binnamangala, S., … Clark, L. (2013). Opioidergic and dopaminergic manipulation of gambling tendencies: a preliminary study in male recreational gamblers. *Frontiers in Behavioral Neuroscience*, *7*:138. 10.3389/fnbeh.2013.00138 PMC3791382 24109443

[CR157] Price DD, Harkins SW, Rafii A, Price C (1986). A simultaneous comparison of Fentanylʼs analgesic effects on experimental and clinical pain. Pain.

[CR158] Price RC, Christou NV, Backman SB, Stone L, Schweinhardt P (2016). Opioid-receptor antagonism increases pain and decreases pleasure in obese and non-obese individuals. Psychopharmacology.

[CR159] Primac DW, Mirsky AF, Rosvold HE (1957). Effects of centrally acting drugs on two tests of brain damage. Archives of Neurology And Psychiatry.

[CR160] Prossin AR, Koch AE, Campbell PL, Barichello T, Zalcman SS, Zubieta JK (2015). Acute experimental changes in mood state regulate immune function in relation to central opioid neurotransmission: a model of human CNS-peripheral inflammatory interaction. Molecular Psychiatry.

[CR161] Quednow BB, Csomor PA, Chmiel J, Beck T, Vollenweider FX (2008). Sensorimotor gating and attentional set-shifting are improved by the μ-opioid receptor agonist morphine in healthy human volunteers. International Journal of Neuropsychopharmacology.

[CR162] Rabiner EA, Beaver J, Makwana A, Searle G, Long C, Nathan PJ (2011). Pharmacological differentiation of opioid receptor antagonists by molecular and functional imaging of target occupancy and food reward-related brain activation in humans. Molecular Psychiatry.

[CR163] Redpath J, Pleuvry B (1982). Double-blind comparison of the respiratory and sedative effects of codeine phosphate and (+/-)-glaucine phosphate in human volunteers. British Journal of Clinical Pharmacology.

[CR164] Ribas-Fernandes JJF, Solway A, Diuk C, McGuire JT, Barto AG, Niv Y, Botvinick MM (2011). A neural signature of hierarchical reinforcement learning. Neuron.

[CR165] Robbins TW, Arnsten AFT (2009). The neuropsychopharmacology of fronto-executive function: Monoaminergic modulation. Annual Review of Neuroscience.

[CR166] Roche DJO, Worley MJ, Courtney KE, Bujarski S, London ED, Shoptaw S, Ray LA (2017). Naltrexone moderates the relationship between cue-induced craving and subjective response to methamphetamine in individuals with methamphetamine use disorder. Psychopharmacology.

[CR167] Rowland NE, Mukherjee M, Robertson K (2001). Effects of the cannabinoid receptor antagonist SR 141716, alone and in combination with dexfenfluramine or naloxone, on food intake in rats. Psychopharmacology.

[CR168] Rukstalis M, Jepson C, Strasser A, Lynch KG, Perkins K, Patterson F, Lerman C (2005). Naltrexone reduces the relative reinforcing value of nicotine in a cigarette smoking choice paradigm. Psychopharmacology.

[CR169] Russell JA (2003). Core affect and the psychological construction of emotion. Psychological Review.

[CR170] Saanijoki, T., Tuominen, L., Tuulari, J. J., Nummenmaa, L., Arponen, E., Kalliokoski, K., & Hirvonen, J. (2018). Opioid release after high-intensity interval training in healthy human subjects. *Neuropsychopharmacology*, *43*, 246–254. 10.1038/npp.2017.148 PMC5729560 28722022

[CR171] Saarialho-Kere U (1988). Psychomotor, respiratory and neuroendocrinological effects of nalbuphine and haloperidol, alone and in combination, in healthy subjects. British Journal of Clinical Pharmacology.

[CR172] Saarialho-Kere U, Mattila MJ, Paloheimo M, Seppälä T (1987). Psychomotor, respiratory and neuroendocrinological effects of buprenorphine and amitriptyline in healthy volunteers. European Journal of Clinical Pharmacology.

[CR173] Saarialho-Kere U, Mattila MJ, Seppälä T (1986). Pentazocine and codeine: effects on human performance and mood and interactions with diazepam. Medical Biology.

[CR174] Saarialho-Kere U, Mattila MJ, Seppälä T (1988). Parenteral pentazocine: Effects on psychomotor skills and respiration, and interactions with amitriptyline. European Journal of Clinical Pharmacology.

[CR175] Saarialho-Kere U, Mattila MJ, Seppälä T (1989). Psychomotor, respiratory and neuroendocrinological effects of a μ-opioid receptor agonist (oxycodone) in healthy volunteers. Pharmacology & Toxicology.

[CR176] Saunders B, Inzlicht M, Braver TS (2015). Vigour and fatigue: How variation in affect underlies effective self-control. Motivation and cognitive control.

[CR177] Schoell ED, Bingel U, Eippert F, Yacubian J, Christiansen K, Andresen H (2010). The Effect of Opioid Receptor Blockade on the Neural Processing of Thermal Stimuli. PLoS ONE.

[CR178] Shenhav A, Botvinick MM, Cohen JD (2013). The expected value of control: an integrative theory of anterior cingulate cortex function. Neuron.

[CR179] Shenhav, A., Musslick, S., Lieder, F., Kool, W., Griffiths, T. L. L., Cohen, J. D. D., & Botvinick, M. M. (2017). Toward a rational and mechanistic account of mental effort. *Annual Review of Neuroscience*, *40*, 99–124. 10.1146/annurev-neuro-072116-031526 28375769

[CR180] Shields GS, Sazma MA, Yonelinas AP (2016). The effects of acute stress on core executive functions: A meta-analysis and comparison with cortisol. Neuroscience and Biobehavioral Reviews.

[CR181] Smith GM, Semke CW, Beecher HK (1962). Objective evidence of mental effects of heroin, morphine and placebo in normal subjects. J Pharmacol Exp Ther.

[CR182] Smith KS, Berridge KC (2007). Opioid limbic circuit for reward: interaction between hedonic hotspots of nucleus accumbens and ventral pallidum. Journal of Neuroscience.

[CR183] Solinas M, Goldberg SR (2005). Motivational effects of cannabinoids and opioids on food reinforcement depend on simultaneous activation of cannabinoid and opioid systems. Neuropsychopharmacology.

[CR184] Spruit, I. M., Wilderjans, T. F. T. M., & van Steenbergen, H. (2018). Heart work after errors: Behavioral adjustment following error commission involves cardiac effort. *Cognitive Affective & Behavioral Neuroscience, 18,* 375–388. 10.3758/s13415-018-0576-6 PMC5889424 29464553

[CR185] Stanciu CN, Glass OM, Penders TM (2017). Use of buprenorphine in treatment of refractory depression—a review of current literature. Asian Journal of Psychiatry.

[CR186] Stotts AL, Dodrill CL, Kosten TR (2009). Opioid dependence treatment: options in pharmacotherapy. Expert Opin Pharmacother.

[CR187] Strand MC, Arnestad M, Fjeld B, Mørland J (2017). Acute impairing effects of morphine related to driving: A systematic review of experimental studies to define blood morphine concentrations related to impairment in opioid-naïve subjects. Traffic Injury Prevention.

[CR188] Streufert, S., & Gengo, F. M. (1993). Drugs and behavior: An introduction. In *Effects of drugs on human functioning* (Vol. 9, pp. 1–12). Karger Publishers.

[CR189] Syal S, Ipser J, Terburg D, Solms M, Panksepp J, Malcolm-Smith S (2015). Improved memory for reward cues following acute buprenorphine administration in humans. Psychoneuroendocrinology.

[CR190] Székely JI, Török K, Karczag I, Tolna J, Till M (1986). Effects of D-Met2, Pro5-enkephalinamide on pain tolerance and some cognitive functions in man. Psychopharmacology.

[CR191] Tanum L, Solli KK, Benth JŠ, Opheim A, Sharma-Haase K, Krajci P, Kunøe N (2017). Effectiveness of injectable extended-release naltrexone vs daily buprenorphine-naloxone for opioid dependence: a randomized clinical noninferiority trial. JAMA Psychiatry.

[CR192] Thayer RE (1989). The biopsychology of mood and activation.

[CR193] Treadway MT, Bossaller NA, Shelton RC, Zald DH (2012). Effort-based decision-making in major depressive disorder: a translational model of motivational anhedonia. Journal of Abnormal Psychology.

[CR194] Valentino RJ, Van Bockstaele E (2015). Endogenous opioids: The downside of opposing stress. Neurobiology of Stress.

[CR195] van der Wel, P., & van Steenbergen, H. (2018). Pupil dilation as an index of effort in cognitive control tasks: A review. *Psychonomic Bulletin and Review, 25,* 2005–2015. 10.3758/s13423-018-1432-y PMC6267528 29435963

[CR196] van Steenbergen H, Gendolla GHE, Tops M, Koole SL (2015). Affective modulation of cognitive control: A biobehavioral perspective. Handbook of Biobehavioral Approaches to Self- ….

[CR197] van Steenbergen, H., Band, G. P. H., & Hommel, B. (2009). Reward counteracts conflict adaptation: evidence for a role of affect in executive control. *Psychological Science*, *20*, 1473–1477. 10.1111/j.1467-9280.2009.02470.x 19906127

[CR198] van Steenbergen H, Band GPH, Hommel B (2010). In the mood for adaptation. Psychological Science.

[CR199] van Steenbergen, H., Band, G. P. H., & Hommel, B. (2015). Does conflict help or hurt cognitive control? Initial evidence for an inverted U-shape relationship between perceived task difficulty and conflict adaptation. *Frontiers in Psychology*, *6*. 10.3389/fpsyg.2015.00974 PMC4498021 26217287

[CR200] Van Steenbergen H, Band GPH, Hommel B, Rombouts SARB, Nieuwenhuis S (2015). Hedonic hotspots regulate cingulate-driven adaptation to cognitive demands. Cerebral Cortex.

[CR201] van Steenbergen H, Weissman DH, Stein DJ, Malcolm-Smith S, van Honk J (2017). More pain, more gain: Blocking the opioid system boosts adaptive cognitive control. Psychoneuroendocrinology.

[CR202] Verster JC, Veldhuijzen DS, Volkerts ER (2006). Effects of an opioid (oxycodone/paracetamol) and an NSAID (bromfenac) on driving ability, memory functioning, psychomotor performance, pupil size, and mood. Clinical Journal of Pain.

[CR203] Veselis RA, Reinsel RA, Feshchenko VA, Wronski M, Dnistrian A, Dutchers S, Wilson R (1994). Impaired memory and behavioral performance with fentanyl at low plasma concentrations. Anesthesia and Analgesia.

[CR204] Vinckier F, Rigoux L, Oudiette D, Pessiglione M (2018). Neuro-computational account of how mood fluctuations arise and affect decision making. Nature Communications.

[CR205] Volavka J, Dornbush R, Mallya A, Cho D (1979). Naloxone fails to affect short-term memory in man. Psychiatry Research.

[CR206] Walker DJ, Zacny JP (1998). Subjective, psychomotor, and analgesic effects of oral codeine and morphine in healthy volunteers. Psychopharmacology.

[CR207] Walker DJ, Zacny JP (1999). Subjective, psychomotor, and physiological effects of cumulative doses of opioid mu agonists in healthy volunteers. The Journal of Pharmacology and Experimental Therapeutics.

[CR208] Walker DJ, Zacny JP, Galva KE, Lichtor JL (2001). Subjective, psychomotor, and physiological effects of cumulative doses of mixed-action opioids in healthy volunteers. Psychopharmacology.

[CR209] Wanigasekera V, Lee MC, Rogers R, Kong Y, Leknes S, Andersson J, Tracey I (2012). Baseline reward circuitry activity and trait reward responsiveness predict expression of opioid analgesia in healthy subjects. Proceedings of the National Academy of Sciences of the United States of America.

[CR210] Wardle MC, Bershad AK, de Wit H (2016). Naltrexone alters the processing of social and emotional stimuli in healthy adults. Social Neuroscience.

[CR211] Wassum KM, Cely IC, Balleine BW, Maidment NT (2011). μ-opioid receptor activation in the basolateral amygdala mediates the learning of increases but not decreases in the incentive value of a food reward. Journal of Neuroscience.

[CR212] Wassum KM, Cely IC, Maidment NT, Balleine BW (2009). Disruption of endogenous opioid activity during instrumental learning enhances habit acquisition. Neuroscience.

[CR213] Wassum KM, Ostlund SB, Maidment NT, Balleine BW (2009). Distinct opioid circuits determine the palatability and the desirability of rewarding events. Proceedings of the National Academy of Sciences of the United States of America.

[CR214] Weber SC, Beck-Schimmer B, Kajdi M-E, Müller D, Tobler PN, Quednow BB (2016). Dopamine D2/3- and μ-opioid receptor antagonists reduce cue-induced responding and reward impulsivity in humans. Translational Psychiatry.

[CR215] Wechsler D (2014). Wechsler adult intelligence scale–Fourth Edition (WAIS–IV).

[CR216] Weerts EM, McCaul ME, Kuwabara H, Yang X, Xu X, Dannals RF (2013). Influence of OPRM1 Asn40Asp variant (A118G) on [ 11C] carfentanil binding potential: Preliminary findings in human subjects. International Journal of Neuropsychopharmacology.

[CR217] Yarkoni T, Poldrack RA, Nichols TE, Van Essen DC, Wager TD (2011). Large-scale automated synthesis of human functional neuroimaging data. Nature Methods.

[CR218] Yeomans MR (1995). Opioids and human food choice. Appetite.

[CR219] Yeomans MR, Gray RW (2002). Opioid peptides and the control of human ingestive behaviour. Neuroscience and Biobehavioral Reviews.

[CR220] Yik MSM, Russell JA, Barrett LF (1999). Structure of self-reported current affect: Integration and beyond. Journal of Personality and Social Psychology.

[CR221] Yovell Y, Bar G, Mashiah M, Baruch Y, Briskman I, Asherov J (2016). Ultra-low-dose buprenorphine as a time-limited treatment for severe suicidal ideation: A randomized controlled trial. American Journal of Psychiatry.

[CR222] Zacny JP (1995). A review of the effects of opioids on psychomotor and cognitive functioning in humans. Experimental and Clinical Psychopharmacology.

[CR223] Zacny JP (2003). Characterizing the subjective, psychomotor, and physiological effects of a hydrocodone combination product (Hycodan) in non-drug-abusing volunteers. Psychopharmacology.

[CR224] Zacny JP, Coalson DW, Lichtor JL, Yajnik S, Thapar P (1994). Effects of naloxene on the subjective and psychomotor effects of nitrous oxide in humans. Pharmacology, Biochemistry and Behavior.

[CR225] Zacny JP, Conley K, Galinkin J (1997). Comparing the subjective, psychomotor and physiological effects of intravenous buprenorphine and morphine in healthy volunteers. Journal of Pharmacology and Experimental Therapeutics.

[CR226] Zacny JP, Conley K, Marks S (1997). Comparing the Subjective, Psychomotor and Physiological Effects of Intravenous Nalbuphine and Morphine in Healthy Volunteers. The Journal of Pharmacology and Experimental Therapeutics.

[CR227] Zacny JP, de Wit H (2009). The prescription opioid, oxycodone, does not alter behavioral measures of impulsivity in healthy volunteers. Pharmacology Biochemistry and Behavior.

[CR228] Zacny JP, Goldman RE (2004). Characterizing the subjective, psychomotor, and physiological effects of oral propoxyphene in non-drug-abusing volunteers. Drug and Alcohol Dependence.

[CR229] Zacny JP, Gutierrez S (2003). Characterizing the subjective, psychomotor, and physiological effects of oral oxycodone in non-drug-abusing volunteers. Psychopharmacology.

[CR230] Zacny JP, Gutierrez S (2008). Subjective, psychomotor, and physiological effects profile of hydrocodone/acetaminophen and oxycodone/acetaminophen combination products. Pain Medicine.

[CR231] Zacny JP, Gutierrez S (2009). Within-subject comparison of the psychopharmacological profiles of oral hydrocodone and oxycodone combination products in non-drug-abusing volunteers. Drug and Alcohol Dependence.

[CR232] Zacny JP, Gutierrez S (2011). Subjective, psychomotor, and physiological effects of oxycodone alone and in combination with ethanol in healthy volunteers. Psychopharmacology.

[CR233] Zacny JP, Hill JL, Black ML, Sadeghi P (1998). Comparing the subjective, psychomotor and physiological effects of intravenous pentazocine and morphine in normal volunteers. The Journal of Pharmacology and Experimental Therapeutics.

[CR234] Zacny JP, Lance Lichtor J, Binstock W, Coalson DW, Cutter T, Flemming DC, Glosten B (1993). Subjective, behavioral and physiological responses to intravenous meperidine in healthy volunteers. Psychopharmacology.

[CR235] Zacny JP, Lichtor JL, Flemming D, Coalson DW, Thompson WK (1994). A dose-response analysis of the subjective, psychomotor and physiological effects of intravenous morphine in healthy volunteers. Journal of Pharmacology and Experimental Therapeutics.

[CR236] Zacny JP, Lichtor JL, JG Z, de Wit H (1992). Effects of fasting on responses to intravenous fentanyl in healthy volunteers. Journal of Substance Abuse.

[CR237] Zacny JP, Lichtor JL, Klafta JM, Alessi R, Apfelbaum JL (1996). The effects of transnasal butorphanol on mood and psychomotor functioning in healthy volunteers. Anesthesia and Analgesia.

[CR238] Zacny JP, Lichtor JL, Thapar P, Coalson DW, Flemming D, Thompson WK (1994). Comparing the subjective, psychomotor and physiological effects of intravenous butorphanol and morphine in healthy volunteers. Journal of Pharmacology and Experimental Therapeutics.

[CR239] Zacny JP, Lichtor SA (2008). Within-subject comparison of the psychopharmacological profiles of oral oxycodone and oral morphine in non-drug-abusing volunteers. Psychopharmacology.

[CR240] Zacny JP, McKay MA, Toledano AY, Marks S, Young CJ, Klock PA, Apfelbaum JL (1996). The effects of a cold-water immersion stressor on the reinforcing and subjective effects of fentanyl in healthy volunteers. Drug and Alcohol Dependence.

[CR241] Zubieta J-KK, Ketter TA, Bueller JA, Xu YJ, Kilbourn MR, Young EA, Koeppe RA (2003). Regulation of human affective responses by anterior cingulate and limbic mu-opioid neurotransmission. Archives of General Psychiatry.

